# Developing a Chitosan/polyvinyl alcohol hydrogel for gastro-retentive release of ranitidine and enhanced anti-ulcerative properties

**DOI:** 10.1186/s12896-025-01040-x

**Published:** 2025-09-10

**Authors:** Shimaa A. Sadek, Sohair R. Fahmy, Sara Bayoumi Ali, Marwa Ahmed Abdelfattah, Ahmed Mostafa Fahmy, Kirolos R. Mansour, Haneen Abdullah, Yousef Mohamed, Nada Hazem, Arsany Ehab Fayez, Christine Fouad Nasr, Gana Khaled Desouky, Abdelrahman W. Hassan, Khadiga M. Gaafar

**Affiliations:** 1 Zoology Department, Faculty of Science, Cairo University, Giza, 12613 Egypt; 2 Biotechnology/Biomolecular Chemistry Department, Faculty of Science, Cairo University, Giza, 12613 Egypt

**Keywords:** Ranitidine, Gastro-retentive drug delivery, Hydrogel, Chitosan, Gastric ulcer

## Abstract

Ranitidine is widely used to treat gastrointestinal conditions, but recent studies have revealed severe potential side effects, including a link to cancer. Therefore, this study aims to develop a new gastro-retentive formulation of ranitidine by utilizing the biocompatibility and biodegradability of Chitosan, along with the strength and hydrophilicity of polyvinyl alcohol (PVA). A chitosan/PVA/ranitidine hydrogel was created using the freeze-thaw method and evaluated for stability, ranitidine release behavior, and efficacy in treating ulcers in rats compared to a commercial formulation. The hydrogel demonstrates an average particle size of 69 nm, a polydispersity index of 0.344, and a zeta potential of + 38 mV. Transmission Electron Microscopy confirmed the spherical shape of the formulation, while X-ray diffraction verified its crystalline structure. Additionally, the study observed an impressive encapsulation efficiency of 98.66% ± 1.01 and a high drug content of 49.82% ± 1.29, as confirmed by Fourier transform infrared analysis. The prepared hydrogel controls the release of ranitidine over 12 h, with an average release of 87.98% ± 4.01%. The hydrogel exhibits minimal degradation over 15 days, greater thermal stability than ranitidine, and adequate stability in acidic gastric conditions. Furthermore, the cytotoxicity assay demonstrated that the hydrogel is biocompatible and promotes cell growth. The study discovered that the hydrogel formulation enhances the effects of ranitidine, particularly its antioxidant and anti-inflammatory properties. In vivo studies illustrated the hydrogel’s promising ulcer-healing properties, suggesting potential use in treating peptic ulcers. Hence, the chitosan/PVA hydrogel can be used as a possible drug delivery system for the sustained release of ranitidine.

## Introduction

Ranitidine (Zantac^®^) is a selective antagonist of histamine H_2_ receptors on gastric parietal cells. It has been extensively utilized in treating conditions such as peptic ulcers, gastroesophageal reflux disease (GERD), and hyperacidity-related disorders by suppressing gastric acid secretion [1]. Ranitidine treats peptic ulcers by blocking histamine from binding to H_2_ histamine receptors in the stomach, which reduces stomach acid. Until recently, Zantac^®^ was a popular over-the-counter H_2_-antagonist brand and consistently ranked among the top 20 prescribed drugs in several countries. Recent findings have raised concerns about the safety of ranitidine, revealing that under acidic gastric conditions, the drug’s amine groups may interact with nitrites to produce N-nitrosodimethylamine (NDMA), a compound classified as a probable human carcinogen. This prompted regulatory bodies, including the FDA, to investigate and issue safety warnings [2, 3]. Following this discovery, the US Food and Drug Administration (FDA) issued a warning about the potential risks, including the risk of cancer from exposure to NDMA [4]. According to the FDA, NDMA levels in ranitidine increase during standard storage and rise significantly when exposed to higher temperatures, which may occur during shipping and handling [5]. Moreover, ranitidine demonstrates limited oral bioavailability, which is attributable to inadequate absorption or degradation within the gastrointestinal (GI) tract [6]. Rapid gastrointestinal transit can lead to incomplete drug release, diminishing the dose’s effectiveness [7]. As a result, the researcher focused on creating a new formula for ranitidine to decrease its interaction with nitrite in the stomach, reduce the production of NDMA, and improve its bioavailability. Additionally, the ranitidine’s short biological half-life of approximately 2.5 to 3 h and absolute bioavailability of 50% also support the development of a sustained-release formulation [6].

Gastroretentive drug delivery systems (GRDDS) have emerged as a promising strategy to overcome challenges related to drugs with narrow absorption windows, low stability in alkaline conditions, or high solubility in acidic environments [8]. These systems extend gastric residence time, thereby enhancing absorption and clinical efficacy. They improve the sustained release of orally administered drugs and prevent rapid gastric secretion to decrease adverse drug reactions [9]. Moreover, these systems continuously release the drug before it reaches the absorption zone, ensuring it is optimally bioavailable [10]. There are several formulations to prolong gastric retention time, including swelling and expanding systems, polymeric bioadhesive systems, and other devices for delayed gastric emptying [11].

Mucoadhesive drug delivery systems have attracted interest due to their ability to adhere to the gastrointestinal mucosa, promoting localized and prolonged drug release at the site of action [12]. Hydrogels are hydrophilic polymers with a cross-linked network structure that can adhere to mucus membranes [13]. Pharmaceuticals use hydrogels as carriers for controlled drug delivery, with release controlled by swelling and biodegradation properties. The swelling properties of hydrogels are primarily associated with the elasticity of the network, the existence of hydrophilic functional groups in the polymer chains, the degree of cross-linking, and the porosity of the polymer [14]. Various polymers, including poly-lactic acid (PLA), Carbopol, poly (acrylic acid), chitosan, and polyethylene glycol (PEG), can be used to fabricate mucoadhesive hydrogels [15].

Chitosan is a bioadhesive polysaccharide produced by deacetylating chitin in the presence of an alkaline substance. It is extensively studied as a promising drug delivery system due to its unique properties, exceptionally high bioadhesivity, proper viscosity, non-toxicity, low cost, excellent biocompatibility, bioactivity, and biodegradability [16]. Further improvements in the mechanical properties of chitosan-based hydrogel have been demonstrated by incorporating hydrophilic polyvinyl alcohol (PVA) during its preparation [17]. Polyvinyl alcohol (PVA) is a versatile synthetic polymer crucial in drug delivery systems [18]. Its hydrophilicity and mechanical strength make it suitable for drug encapsulation, controlled release, and the formation of structures like hydrogels, which improve therapeutic efficacy and minimize side effects [19].

The current research aimed to tackle challenges associated with ranitidine by developing mucoadhesive/gastro-retentive delivery systems using chitosan-based hydrogel for ranitidine. This formulation is intended to improve the drug’s performance in the stomach, enhancing its stability and reducing potential side effects, ultimately improving its biopharmaceutical performance. To achieve this, ranitidine was combined with chitosan/PVA hydrogel using the freeze-thaw method. Its stability and swelling behaviors were then evaluated to assess its potential for gastric retention systems. The new ranitidine formulation was also compared with its commercial counterpart for biological activities such as antioxidant, anti-inflammatory, and analgesic effects. Furthermore, the study assessed the anti-ulcerogenic efficacy of the new ranitidine formulation, chitosan/PVA/Ranitidine hydrogel, in rats.

## Materials and methods

### Chemicals and reagents

Chitosan (low molecular weight, 75–80% deacetylated) from Alamia Company for Chemicals (Mohy El-Din Abou El-Ezz St., Giza, Egypt), Indomethacin, polyvinyl alcohol (PVA), and 2,2-Diphenyl-1-picrylhydrazyl (DPPH) were obtained from Sigma Chemical Co. (St. Louis, MO. USA). Ranitidine (Zantac^®^, 150 mg) was obtained from the local pharmacy (Giza, Egypt). The commercial kits for all biochemical parameters were purchased from Biodiagnostic Company (Dokki, Giza, Egypt). All reagents utilized in the experiment were of analytical grade.

### Experimental animals

For the preliminary analgesic activity, this study used adult Swiss male albino mice (Mus musculus) weighing 20–25 g. The main experiment involved adult Wistar rats (Rattus norvegicus) weighing 150–170 g. These animals were obtained from the National Research Center (NRC) in Egypt and housed in polypropylene cages with five animals each. They were kept in a well-ventilated facility at 23 ± 2 °C on a natural day/night cycle. The animals received standard chow pellets and water ad libitum. Before the study, they acclimatized to the conditions for seven days. The experimental protocols were approved by the Cairo University Institutional Animal Care and Use Committee (IACUC) in Egypt (Approval no: CU/I/F/5/24). All procedures complied with the Guide for the Care and Use of Laboratory Animals, 8th Edition, 2011 (the Guide).

### Chitosan/polyvinyl alcohol hydrogel preparation

The Chitosan/polyvinyl alcohol hydrogel was prepared using a freeze-thaw method, enabling physical crosslinking, as previously described [20]. Initially, 2% w/v chitosan was dissolved in 1% acetic acid at 37 °C with vigorous stirring at 800 rpm until a clear solution was obtained. A 1% w/v PVA solution was prepared by dissolving PVA in deionized water at 90 °C, stirring at 600 rpm for 1 h. Two polymer solutions were mixed in a 1:1 volume ratio (v/v), equating to a 1:1 mass ratio of chitosan to PVA. The mixture was stirred at 250 rpm for 30 min at 40 °C to obtain a homogeneous polymer blend. The polymer solution was divided into two parts: one was used to incorporate ranitidine, and the other formed a chitosan/PVA hydrogel through four freeze-thaw cycles (12 h freezing at − 20 °C, 12 h thawing at room temperature), followed by freeze-drying at − 50 °C under vacuum for 48 h. The final mixture underwent these cycles to promote physical crosslinking, resulting in a porous structure.

### Incorporation of ranitidine into chitosan/polyvinyl alcohol hydrogel (Chitosan/PVA/Ranitidine hydrogel)

First, a 5% w/v Ranitidine solution was prepared in 5% DMSO and stirred using a magnetic stirrer for 45 min, with the rotation speed gradually increasing from 300 rpm to 600 rpm to ensure complete mixing and dissolution. This drug solution was then added dropwise to the polymer mixture while continuously stirred at 800 rpm for one hour to obtain a uniform distribution. The final mixture underwent four freeze–thaw cycles to promote physical crosslinking. Each cycle involved freezing at − 20 °C for 12 h and thawing at room temperature for 12 h. After completing all cycles, the hydrogels were freeze-dried at − 50 °C under vacuum for 48 h to obtain the final porous structure.

### Physicochemical characterization of Chitosan/PVA/Ranitidine hydrogel

The hydrogel’s shape, pore sizes, and distribution are studied using Transmission Electron Microscopy (TEM) in the Electron Microscopy Unit at the Faculty of Agriculture, Cairo University. The process utilized TEM at an accelerated voltage of 120 kV (JEM- JEM 2100 F; JEOL Ltd, Tokyo, Japan) based on a previously described protocol [21]. The hydrogel’s average hydrodynamic size and zeta potential were also determined using dynamic light scattering (DLS) (Zetasizer Nano Series, Malvern, UK). Additionally, the optical absorption of the Chitosan/PVA/Ranitidine hydrogel was measured using a double-beam ultraviolet-visible (UV-Vis) spectrophotometer (Shimadzu 1801, Kyoto 604–8511, Japan) at wavelengths between 200 and 800 nm, at room temperature.

### Structure characterization of Chitosan/PVA/Ranitidine hydrogel

The chemical interaction between ranitidine and other hydrogel ingredients was studied by identifying their functional groups through Fourier transform infrared spectroscopy (FT-IR). The Chitosan/PVA/Ranitidine hydrogel underwent freeze-drying, was combined with potassium bromide (KBr) pellet, and then pressed into disks. An FT-IR spectrophotometer (JASCO FTIR-6200, JASCO International Co., Ltd- Japan) was utilized to record IR spectra in the 4000–400 cm^-1^ [22]. Understanding the crystalline structure of a sample is essential for precise material analysis and synthesis. This is achieved through X-ray diffraction (XRD) using an X-ray diffractometer (Reguka Miniflex, India) with Cu Kα radiation, operating at 30 kV/15 mA. All measurements were achieved at room temperature within the diffraction angle 2θ range of 0–80° and at a speed of 1° per minute. Also, XRD was used to estimate the average crystallite size of Chitosan/PVA/Ranitidine hydrogel using Debye–Scherrer’s formula:

$$D{\rm{ }} = {\rm{ }}0.9\lambda {\rm{ }}/{\rm{ }}\beta {\rm{ }}cos{\rm{ }}\theta $$

### Determination of encapsulation efficiency (EE%)

The efficiency of hydrogel in trapping Ranitidine was assessed based on the method described by Nochos et al. [23]. At first, a Chitosan/PVA/Ranitidine hydrogel weighing 100 mg was submerged in 30 ml of phosphate buffer (pH 1.5) for 4 h, with constant stirring. The mixture was thoroughly mixed and then sonicated to remove any trapped air bubbles, followed by centrifugation at 10,000 rpm for 10 min. Next, the absorbance of free Ranitidine in the supernatant was measured using a UV–Visible spectrophotometer (U-2001, model 121 0032 Hitachi, Tokyo, Japan) at 320 nm, corresponding to the maximum absorption peak of Ranitidine. The following equation was used to calculate the drug encapsulation efficiency (EE%) [24]:

$$EE\% {\rm{ }} = \left( {{C_1} - {C_2}} \right)/{\rm{ }}{C_1} \times {\rm{ }}100$$

C_1_: Total Ranitidine concentration; C_2_: Free Ranitidine concentration.

### Drug content analysis

According to Ullah et al.‘s method, a drug content analysis was performed to quantify the amount of Ranitidine in the hydrogel [22]. 100 mg Ranitidine-loaded hydrogel was placed in 30 ml of phosphate buffer at a pH of 1.5 and stirred for 2 h. Following this, the mixture was centrifuged at 5000 rpm for 30 min. After centrifugation, the sediment was dissolved in 0.1 N HCl and analyzed on a UV-visible spectrophotometer at 320 nm (λmax of Ranitidine) to obtain the entrapped Ranitidine concentration. The Ranitidine content of the hydrogel was calculated with the following equation:

$$\eqalign{& Drug\,{\rm{ }}loading\,{\rm{ }}content{\rm{ }}\left( \% \right) = \cr & \,\,\,\,\,\,\,\,\,\,\,{{Amount\,of\,Ranitidine\,in\,hydrogel} \over {Theorectical\,drug\,content}} \times 100 \cr} $$

### Swelling degree of Chitosan/PVA/Ranitidine hydrogel

The equilibrium swelling ratio (SR) of Ranitidine-loaded hydrogel samples was assessed according to Călina et al. [25]. Briefly, 100 mg of the prepared hydrogel was added to the bicarbonate buffer at different pHs: 5.4, 6.4, and 9.4. Finally, the weight of the samples at various time intervals in triplicate is measured by removing, blotting, weighing, and immersing them again. The swelling ratio was calculated using the following equation:

$$Swelling\,\,\% \% = \left( {{W_s} - {W_d}} \right)/{W_d} \times 100$$

W_s_ is the weight after swelling, and W_d_ is the weight in the dry state of the hydrogel.

### Degradation study of Chitosan/PVA/Ranitidine hydrogel

The Ranitidine-loaded hydrogel was weighed, placed in phosphate buffer (pH 1.5), and incubated at 37 °C. The weight of the hydrogel was recorded on the 1st, 3rd, 7th, and 15th days. The phosphate buffer was refreshed after each measurement at regular intervals. The weight loss (%) at each time interval was calculated using the following equation:

$$Weight\,\,loss{\mkern 1mu} \left( \% \right) = \left( {{W_i} - {W_f}} \right)/{W_f}x100$$

W_i_ is the initial weight of the sample, and W_f_ is the final weight of each sample.

### Moisture retention capacity (MRC) analysis

The MRC of hydrogels was determined by slicing a large piece of Ranitidine-loaded hydrogel into three equal-sized pieces. Each piece was weighed individually, placed in separate petri dishes at room temperature, and then weighed at intervals (2, 4, 6, 8, 10, and 24 h). The MRC was calculated as the water loss rate and the ratio of water held in the hydrogel using a specific equation [26]:

$$MRC\% = \left( {{W_i}/{W_f}} \right){\rm{ }}X100$$

W_i_ is the initial weight, and W_f_ is the weight after a specific time interval.

### In vitro Ranitidine release study

The release of Ranitidine from a hydrogel formulation was tested using the dialysis method described by Salem et al. [27]. Cellulose acetate dialysis bags were submerged in phosphate-buffered saline (PBS) and then filled with 3 mL of a Chitosan/PVA/Ranitidine hydrogel. The bags were placed in 100 mL of PBS at a pH of 1.5, and the solution was stirred at 150 rpm using a magnetic stirrer. At regular intervals, 2 mL samples of the surrounding solution were taken, and the same amount of fresh PBS was added to keep the total volume constant. The absorbance of these samples was measured at 320 nm using a spectrophotometer (U-2001, model 121 0032, Hitachi, Tokyo, Japan). The percentage of Ranitidine released was calculated using established methods. The experiment ended when the Ranitidine concentration in the immersion medium reached a steady state. A similar test was conducted with free Ranitidine at the same concentration. All dissolution experiments were carried out in triplicate. The released drug amount was determined using the following equation:

$$\eqalign{& Amount{\rm{ }}\,of{\rm{ }}\,released{\rm{ }}\,drug{\rm{ }}\left( \% \right){\rm{ }} = \cr & \,\,\,\,{{Amount\,\,of\,\,released\,\,drug\,\,at\,\,specific\,\,time} \over {Amount\,\,of\,\,drug\,\,in\,\,hydrogel}} \times 100 \cr} $$

### Stability studies

#### Thermal stability

Thermogravimetric analysis (TGA) was performed on Ranitidine and Chitosan/PVA/Ranitidine hydrogel using Model-SDTQ600 from the USA. The study was conducted at a heating rate of 10 °C/min under an N_2_ atmosphere with a 100 mL/min purging rate.

#### Physical stability

The physical stability of the Ranitidine hydrogel was evaluated by observing particle size and zeta potential changes after one month at 4 °C. Additionally, the study assessed the hydrogel’s ability to retain Ranitidine during storage by measuring entrapment efficiency and drug content as described.

#### pH effect on Chitosan/PVA/Ranitidine hydrogel stability

Based on the previous method, Chitosan/PVA/Ranitidine hydrogel and Ranitidine stability were assessed at different pH values [28]. The samples were diluted in phosphate-buffered saline at varying pH values and then incubated overnight at 37 °C. Following centrifugation, the concentration of the samples in the supernatant was measured using a UV-Vis spectrophotometer at 320 nm. The results are expressed as a percentage of the soluble fraction relative to the prepared hydrogel diluted in water.

### Assessment of in vitro gastrointestinal behavior of Chitosan/PVA/Ranitidine hydrogel

The simulated gastric and intestinal models were used to study the potential gastrointestinal behavior of the prepared hydrogel based on the adopted protocol [29]. First, simulated gastric fluid (SGF) was prepared by dissolving 2 g of NaCl and 1.4 g of pepsin in H_2_O, adding 7 ml of concentrated HCl, and adjusting the pH to 1.2. For simulated intestinal fluid (SIF), mix 6.8 g of dipotassium phosphate with 190 mL of solution and adjust the pH to 7.4. The Chitosan/PVA/Ranitidine hydrogel was mixed with SGF or SIF in a 1:3 ratio and incubated at 37 °C for 2 h, reflecting the average duration of GI transit. During digestion, a volume of the mixture was withdrawn at intervals (0–120 min) to assess Ranitidine release by determining encapsulation efficiency as described previously.

### Cell viability assessment of Chitosan/PVA/Ranitidine hydrogel

The cytotoxicity of Ranitidine and its hydrogel formulation was assessed on the viability of VERO cells using 3-(4,5-dimethylthiazol-2-yl)-2,5-diphenyltetrazolium bromide (MTT) [30]. Briefly, Vero Cells (CCL-81) were seeded in 96-well plates at a density of 5 × 10^3^ per well and incubated for 24 h in complete DMEM. After that, the cells were treated with 200 µl Ranitidine and its hydrogel formulation and incubated for 24 h at 37 °C. At the end of incubation, MTT reagent (Sigma-Aldrich) dissolved in phenol red-free DMEM was added to cells at 0.5 mg/mL. After 4 h, the formed formazan salts were dissolved with isopropanol containing 0.1 N HCl. The plate was then measured for absorbance at 570 nm using a microplate reader to determine cell viability.

### Evaluation of biological activities of Chitosan/PVA/Ranitidine hydrogel

#### In vitro antioxidant potency assay

The DPPH assay assessed the antioxidant effect of Ranitidine and Chitosan/PVA/Ranitidine hydrogel. Following the protocol by Brand-Williams et al. [31], a 0.1 mM DPPH solution was prepared by dissolving 4 mg of DPPH in 100 ml of methanol. Then, 40 µL of different concentrations of Ranitidine and Chitosan/PVA/Ranitidine hydrogel (100, 200, 300, 400, and 500 µg/ml) were added to separate tubes containing 2.96 mL of DPPH solution. The reaction mixture was incubated in the dark at room temperature for 20 min. The absorbance was then read at 517 nm using a spectrophotometer (Biomed Diagnostics, White City, USA). The percentage of DPPH scavenging activity was calculated using the following formula:

$$\eqalign{& DPPH\,\,{\rm{ }}scavenging\,\,{\rm{ }}activity{\rm{ }} = {\rm{ }} \cr & \,\,\,\,\,\left[ {\left( {{A_{Control}} - {\rm{ }}{A_{Sample\,{\rm{ }}\,or\,{\rm{ }}\,standard}}/{\rm{ }}{A_{Control}}} \right)} \right]{\rm{ }}x{\rm{ }}100 \cr} $$

#### In vitro anti-inflammatory potency assay

The anti-inflammatory properties of Ranitidine and Chitosan/PVA/Ranitidine hydrogel were compared at various concentrations (100–500 µg/ml) following the membrane stabilization assay demonstrated by Gandhidasan [32]. A fresh blood sample was obtained from a rat and centrifuged at 3000 rpm for 10 min, washed three times with an equal volume of saline, and then diluted with saline to achieve a 10% (v/v) suspension. In each tube, 1 ml of Ranitidine or Chitosan/PVA/Ranitidine hydrogel at different concentrations was mixed with 1 ml of RBC suspension, and all tubes were subsequently incubated at 56 °C for 30 min. After cooling, the tubes were centrifuged at 2500 rpm for 5 min, and the supernatant was measured for hemoglobin content at 546 nm using a UV-Vis spectrophotometer. The percentage of RBC membrane stabilization was calculated using the following formula:

$$\eqalign{& \% \,\,{\rm{ }}stabilization{\rm{ }} = {\rm{ }}100{\rm{ }}-{\rm{ }} \cr & \,\,\,\,\,\,\,\,\,\left[ {{\rm{ }}{A_{sample\,\,{\rm{ }}or\,\,{\rm{ }}standard}}/{A_{control}} \times {\rm{ }}100} \right] \cr} $$

### Analgesic potency assay

Two methods assessed the potential peripheral (acetic acid-induced writhing test) and central (hot plate latency assay) analgesics.

#### Assessment of peripheral analgesic potency

The acetic acid-induced writhing assay evaluated the peripheral analgesic efficacy of Chitosan/PVA/Ranitidine Hydrogel and Ranitidine compared to sodium diclofenac, which served as the standard analgesic agent. In summary, mice received an oral administration of either the vehicle, sodium diclofenac (50 mg/kg body weight), Chitosan/PVA/Ranitidine Hydrogel, or Ranitidine at a dosage of 8 mg/kg body weight. Subsequently, the subjects were intraperitoneally injected with 0.1 ml of a 1% acetic acid solution to provoke a writhing response. After five minutes following the acetic acid injection, the mice were placed in an observation enclosure, where the number of writhing movements (defined as the full extension of the hind limb) for each experimental group was recorded over a period of fifteen minutes. The percentage inhibition of writhing frequency in the treated groups, serving as an index of analgesic effectiveness, was computed using the following formula:

$$\% \,\,Inhibition = \left( {{W_c} - {W_t}} \right)/{W_c} \times 100$$

W_c_ = Number of writhing in the control group; W_t_ = Number of writhing in the treated group.

#### Assessment of central analgesic potency

The efficacy of Chitosan/PVA/Ranitidine Hydrogel and Ranitidine in relieving pain in mice was tested using Eddy’s hot plate method. The mice were administered different treatments: vehicle, sodium diclofenac (50 mg/kg body weight), Chitosan/PVA/Ranitidine Hydrogel, and Ranitidine at doses of 8 mg/kg body weight [33]. After 30 min, the mice were placed individually on a hot plate at 55 ± 1 °C to stimulate pain. The reaction time (paw licking or jumping) was recorded for each mouse at time intervals of 30 min, 60 min, and 90 min after administration of all treatments, with a cutoff time of 15 s to prevent tissue damage.

### Evaluation of anti-ulcerogenic potency of Chitosan/PVA/Ranitidine hydrogel

#### Induction of severe gastric ulcers

Before inducing ulcers, rats will be deprived of food, but not water, for 24 h [34]. The ulcer will be induced by a combination of indomethacin and cold stress (IND + CS). Indomethacin (IND) will be administered as a single oral dose (150 mg/kg) dissolved in 5% sodium bicarbonate [35]. Subsequently, the animals will be exposed to cold stress (CS) at 3–5 °C for 2 h.

#### Experimental design and treatments

After 2 h, the animals were randomly divided into 5 groups (six rats/group) as follows:

- **Group 1** served as control; normal rats were orally administered a single dose of 5% DMSO.
- **Group 2**: Ulcerated rats were orally administered a single dose of 5% DMSO.
- **Group 3**: Ulcerated rats were orally administered a single dose of Ranitidine (30 mg/kg body weight) [36].
- **Group 4**: Ulcerated rats were orally administered a single dose of free hydrogel.
- **Group 5**: Ulcerated rats were orally administered a single dose of Chitosan/PVA/Ranitidine hydrogel (30 mg/kg body weight).

#### Animals handling

One hour after administering all treatments, all the rats underwent behavioral assessments to evaluate their movement and measure anxiety-like behavior. Then, the animals were euthanized under deep anesthesia with sodium pentobarbital. The stomach was removed and immediately blotted with filter paper to remove any traces of blood. The stomach was dissected and cut along the greater curvature, and gastric juice was collected. The mucosa was rinsed with cold normal saline to remove blood contaminants. The number of hemorrhagic and ulcerative lesions in the stomach was counted, and the stomachs of the rats were stored at − 80 °C for biochemical analysis.

#### Behavioral examination using open-field test

The open field was used to evaluate the effects of Ranitidine and its hydrogel formulation on the locomotor and anxiety-like behavior of ulcerated rats. After one hour of treatment, the rats were placed in a 70 × 70 × 35 cm square box with a floor divided into 16 squares for the open field test, and their behavior was observed. Ambulation frequency, rearing frequency, and freezing duration were measured as locomotor and anxiety behavior indices. The box was cleaned with 70% ethanol between tests to prevent olfactory cues.

#### Macroscopic evaluation of gastric mucosal injury

The severity of macroscopic lesions was estimated using an ulcer index on the following scale: 0: Stomachs with no injuries; 0.5: Red coloration; 1.0: Spot ulcers; 1.5: Hemorrhagic streaks; 2.0: Ulcers with an area of > 3 but ≤ 5mm^2^; 3.0: Ulcers > 5mm^2^, the ulcer index was calculated as follows:

$$Ulcer\,\,index{\mkern 1mu} {\mkern 1mu} \left( {UI} \right) = \left[ {{U_N} + {U_S} + {U_P}} \right] \times 10$$

Here, UI represents the ulcer index, U_N_ is the average number of ulcers per animal, U_S_ is the average severity score, and U_P_ is the percentage of animals with an ulcer.

Additionally, the ulcer preventive index was calculated using the following formula, as recommended previously [37]:

$$\eqalign{& Preventive\,\,{\rm{ }}index{\rm{ }} = {\rm{ }}\left[ {U{I_{ulcerated\,\,{\rm{ }}group}} - {\rm{ }}U{I_{treated\,\,{\rm{ }}group}}} \right]/{\rm{ }} \cr & \,\,\,\,\,\,\,\,\,\,\,\,\,\,\,\,\,\,\,\,\,\,\,\,\,\,\,\,\,\,\,\,\,\,\,\,\,\,\,\,\,\,\,\,\,\,\,U{I_{ulcerated{\rm{ }}\,\,group}} \times 100 \cr} $$

#### Analysis of gastric juice (pH, volume, acidity)

##### Determination of gastric pH and volume

Collected gastric contents were placed in centrifuge tubes and centrifuged at 1000 rpm for 10 min at 4 °C. The resulting supernatant was used to measure volume and pH, allowing us to assess gastric mucosal injury.

##### Determination of gastric acidity

First, gastric juice was diluted with distilled water at a ratio of 1:1. Then, the diluted gastric juice was titrated with 0.01 N NaOH using phenolphthalein as an indicator until a permanent pink color appeared [38]. The volume of NaOH used was then recorded. The total acidity, expressed as mEq/L, can be calculated using the following equation:

$$Acidity{\rm{ }}\left( {mEq/L} \right){\rm{ }} = {\rm{ }}\left( \matrix{Vol.{\rm{ }}of{\rm{ }}NaOH{\rm{ }} \times {\rm{ }} \hfill \cr N{\rm{ }} \times {\rm{ }}100 \hfill \cr} \right)/0.1$$

#### Determination of lipid peroxidation, enzymatic and non-enzymatic antioxidant markers

The gastric mucosa was homogenized to 10% (w/v) concentration in an ice-cold Tris-HCl buffer (0.1 M, pH 7.4) and then centrifuged at 3000 rpm for 15 min at 4 °C. The resulting supernatant was used to estimate the levels of malondialdehyde (MDA), reduced glutathione (GSH), catalase (CAT), superoxide dismutase (SOD), and glutathione peroxidase (GPx) using commercially available kits.

#### Histopathological studies

Gastric tissue samples were washed in phosphate-buffered saline and fixed in 10% neutral buffered formalin (pH 7.0) for 24 h. After fixation, the samples were processed to obtain paraffin sections, cut at 5 μm with an optical rotary microtome, stained with hematoxylin-eosin (H&E) and Periodic acid Schiff (PAS), and examined under a light microscope for histological evaluations.

### Data analysis

All data were expressed as mean ± standard error (SEM). One-way analysis of variance (ANOVA) was performed to analyze the significant difference between different groups, followed by the Duncan post hoc test at *P* < 0.05. SPSS software (version 22) was used for statistical analysis.

## Results

### In vitro analysis

#### Characterization of Chitosan/PVA hydrogel containing Ranitidine

##### Average hydrodynamic particle size, zeta potential, and optical absorption

**Fig. 1:**
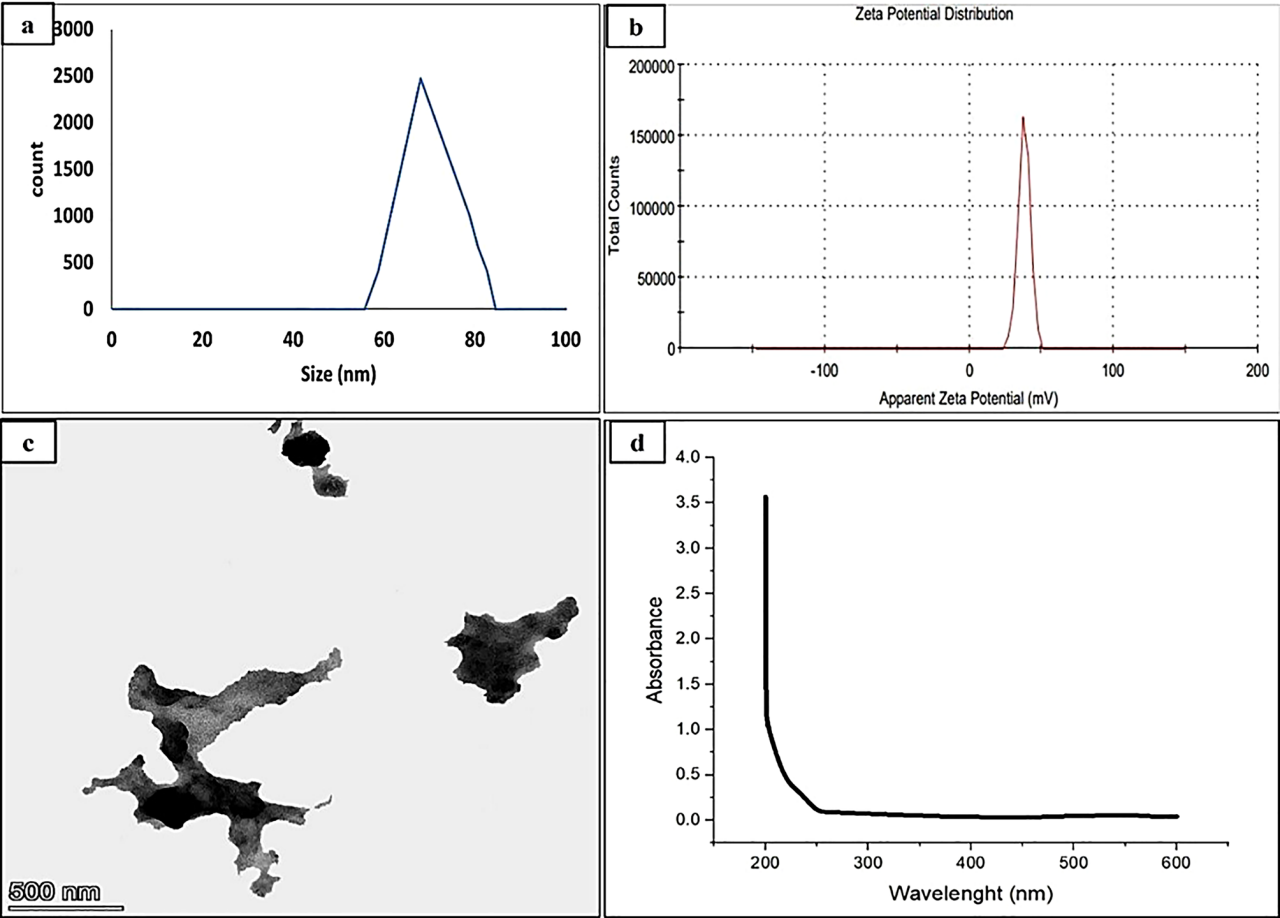
Physicochemical characterization of Chitosan/PVA hydrogel containing ranitidine using dynamic light scattering technique, a transmission electron microscope (TEM) and UV spectra analysis. (**a**) Particle size distribution of Chitosan/PVA hydrogel containing ranitidine using dynamic light scattering technique demonstrating an apparent particle size of 69 nm. (**b**) Zeta potential of Chitosan/PVA hydrogel containing ranitidine. (**c**) TEM image showing the spherical shape of Chitosan/PVA hydrogel containing ranitidine. (**d**) UV–visible absorption spectra of Chitosan/PVA hydrogel containing ranitidine

The hydrogel’s particle distribution showed a single dominant size range, indicating homogenous distribution (Fig. 1a). The Chitosan-based hydrogel-entrapped Ranitidine exhibits an average hydrodynamic size of 69 nm and a PDI of 0.344, indicating its impressive nanoscale dimensions (Fig. 1a). The Chitosan/PVA hydrogel containing Ranitidine demonstrated exceptional stability, as evidenced by its zeta potential of 38 mV, indicating a strong surface electric charge (Fig. 1b). The UV-VIS spectrophotometer detected an absorption characteristic band at 200 nm for the Chitosan/PVA/Ranitidine hydrogel, as shown in Fig. 1d.

##### Morphological surface

Figure 1c shows the transmission electron microscopy (TEM) image of the chitosan/PVA hydrogel loaded with ranitidine, revealing its morphology and particle size. The hydrogel displays mostly spherical nanoparticles with minimum aggregation, indicating a uniform distribution. The average particle size was estimated to be around 55 nm, confirming the nanoscale nature of the hydrogel system. These findings offer valuable insights into the structural characteristics and homogeneity of the chitosan/PVA/Ranitidine formulation.

##### Structure characterization

###### FTIR study

**Fig. 2:**
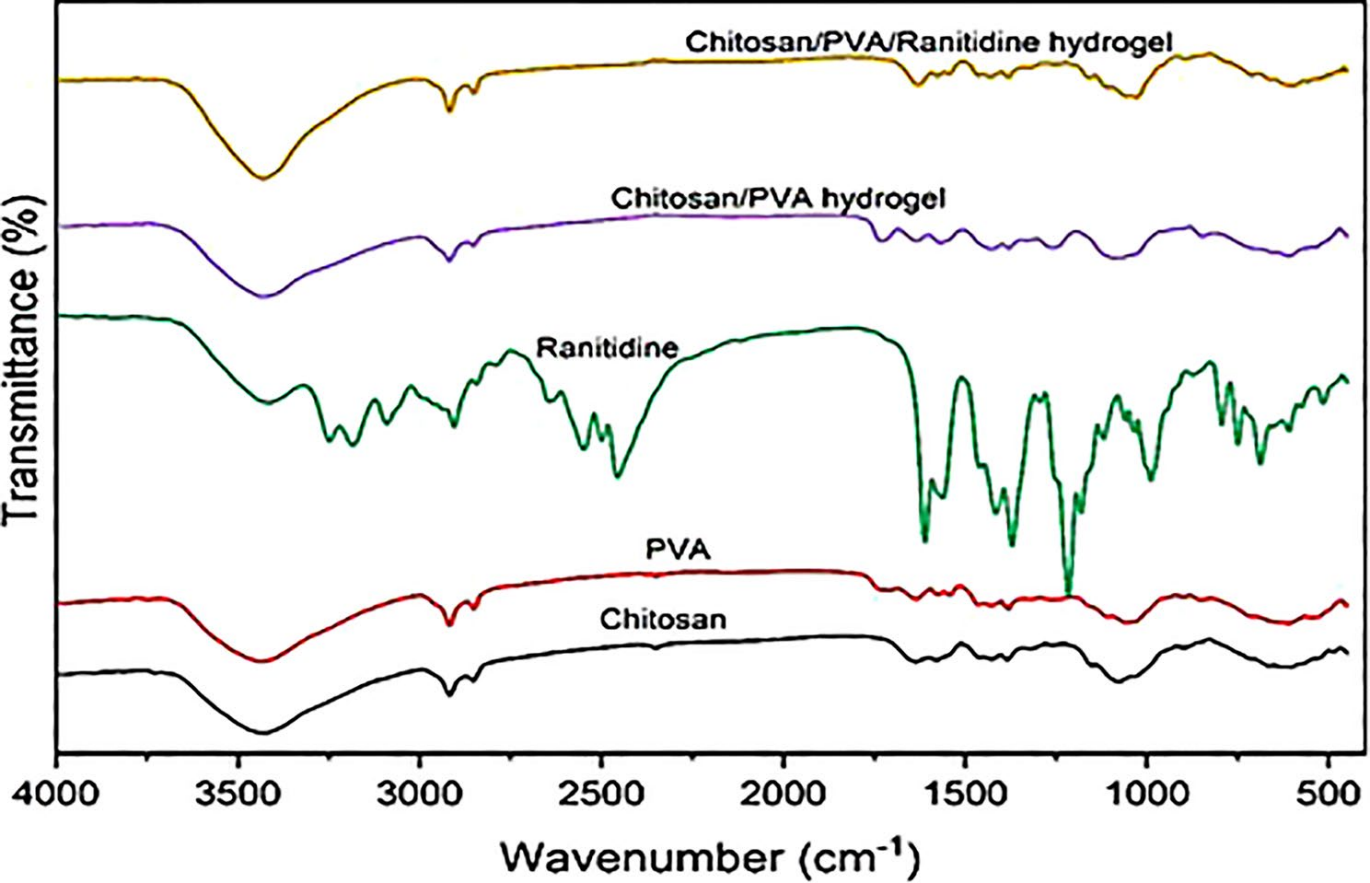
Structural Chitosan/PVA/Ranitidine hydrogel characterization using Fourier transform infrared (FT-IR)

The FT-IR spectrum of the Ranitidine sample shows bands corresponding to the presence of OH, C-H, and C = N chemical groups with characteristics of 3949.5, 2915.84, 1619.91, and 1250 cm^− 1^, respectively (Fig. 2). Chitosan exhibits bands corresponding to OH, CH, and C = C chemical groups with characteristics of 3431.71, 2917.77, and 1636.3 cm^− 1^, respectively. On the other hand, PVA demonstrates characteristic bands at 3744.12, 2918.73, and 1633.41 cm^− 1^ were attributed to OH, CH, and C = C chemical groups. Blending PVA and Chitosan display prominent characteristic bands for O-H, C-H, and C = C chemical groups at 3431.71, 2918.73, and 1634.38 cm-1, respectively, as demonstrated in Fig. 2. Conversely, the Chitosan/PVA/Ranitidine hydrogel exhibits similar bands related to the O-H, C-H, and C = C chemical groups with 3938.89, 2917.77, and 1629.55 cm^− 1^ characteristic values, respectively. These FT-IR results indicate the absence of new band formations, suggesting that Ranitidine has been encapsulated within the Chitosan/PVA hydrogel without forming covalent bonds.

###### XRD study

**Fig. 3:**
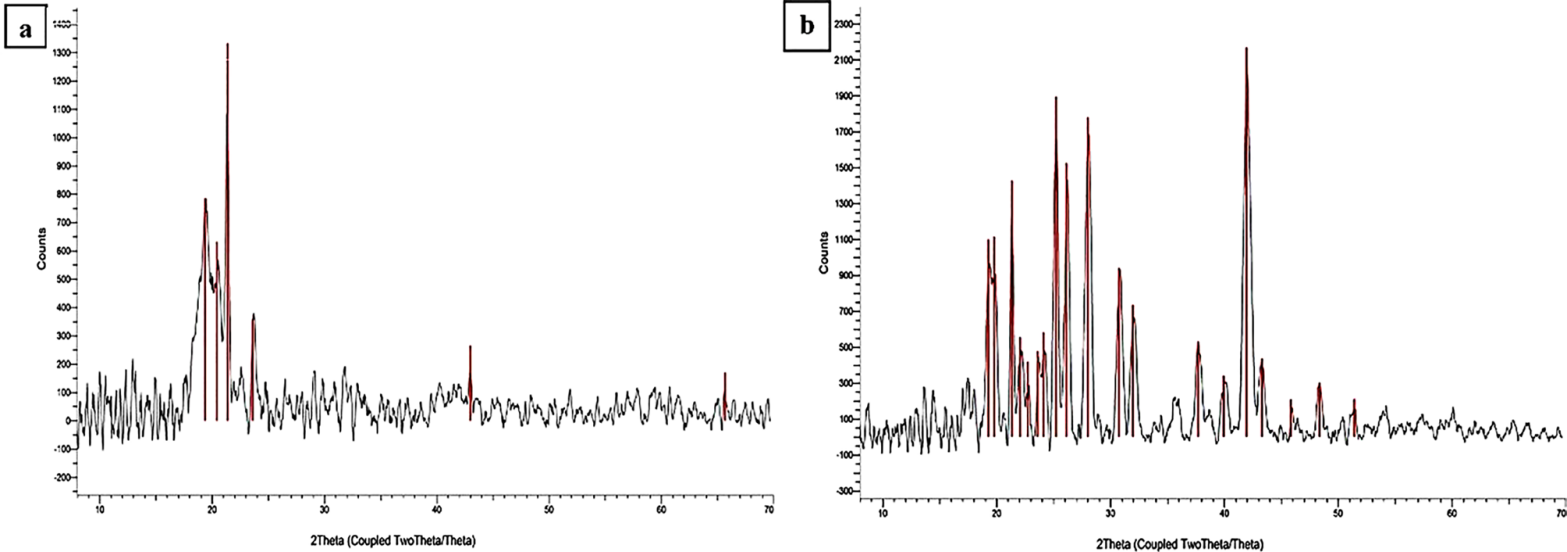
X-ray diffraction pattern for the crystalline structure of (**a**) Ranitidine sample and (**b**) Chitosan/PVA/Ranitidine hydrogel

Figure 3a illustrates the X-ray diffraction (XRD) patterns of pure ranitidine and chitosan/PVA/ranitidine hydrogel samples. The diffraction pattern of ranitidine exhibits multiple peaks spanning from 19° to 65°, with the most prominent peak at 2θ = 21.36°, thereby indicating its crystalline nature. Conversely, the chitosan/PVA/ranitidine hydrogel displays sharp, well-defined peaks between 19° and 51°, with the highest intensity observed at 2θ = 41.94°, similarly confirming crystallinity (Fig. 3b). The application of Scherrer’s equation to the hydrogel sample yielded an average crystallite size of 15.50 nm, further corroborating the existence of nanoscale crystalline domains within the hydrogel matrix.

#### Encapsulation efficiency and drug content

**Fig. 4:**
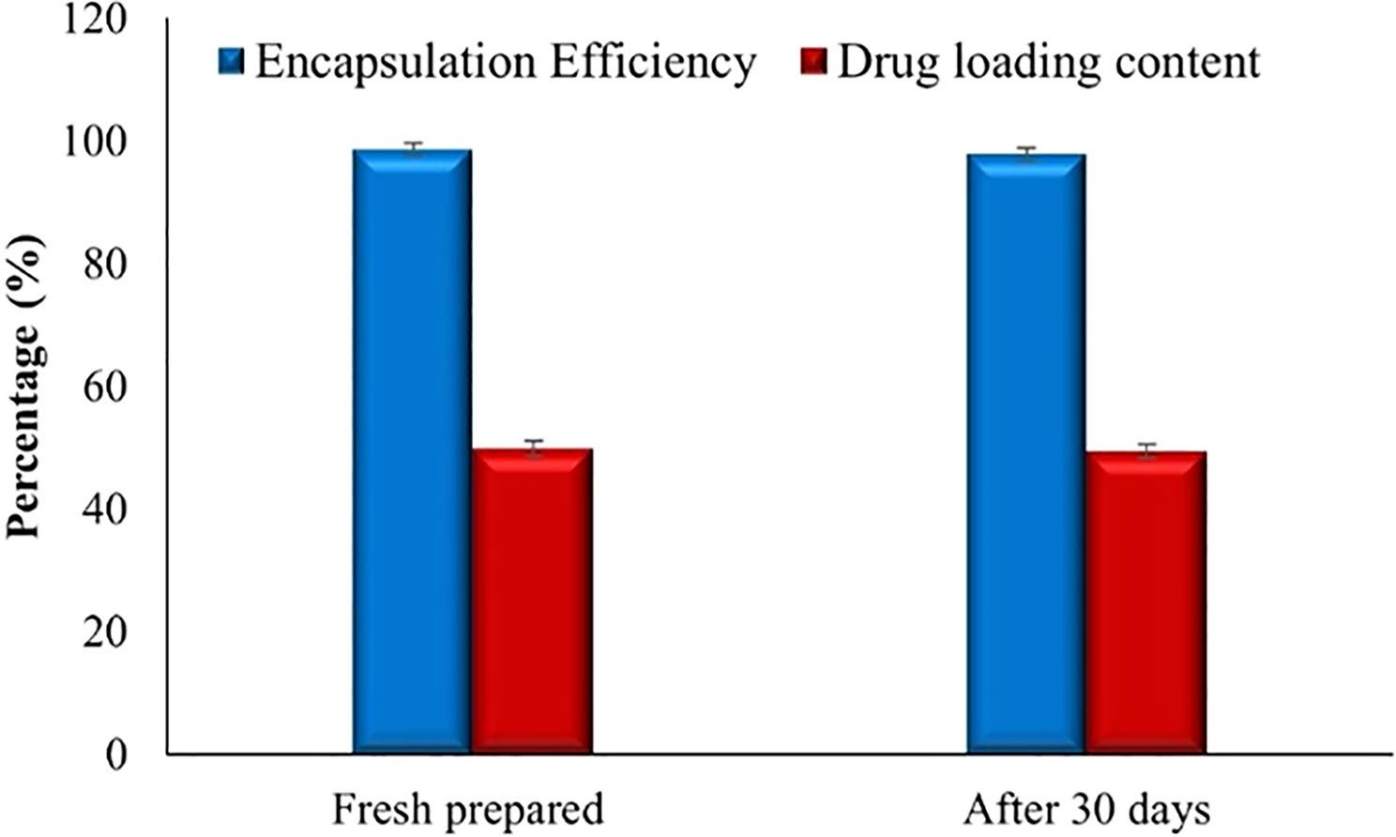
The encapsulation efficiency (EE%) and drug loading capacity (LC%) of Chitosan/PVA hydrogel containing ranitidine. Values are expressed as the mean of three replicate determination ± SEM

Figure 4 illustrates the encapsulation efficiency (EE) and drug loading capacity of the Chitosan/PVA hydrogel containing Ranitidine over 30 days. The EE of the Chitosan/PVA hydrogel with Ranitidine is approximately 98.6 ± 1.01%, remaining consistently high and stable throughout the 30 days, with no significant change in EE percentage. Additionally, drug loading analysis revealed that the hydrogel consistently maintained a loading capacity exceeding 50%, confirming its strong ability to retain and stabilize the incorporated drug over time.

#### Swelling behavior

**Fig. 5:**
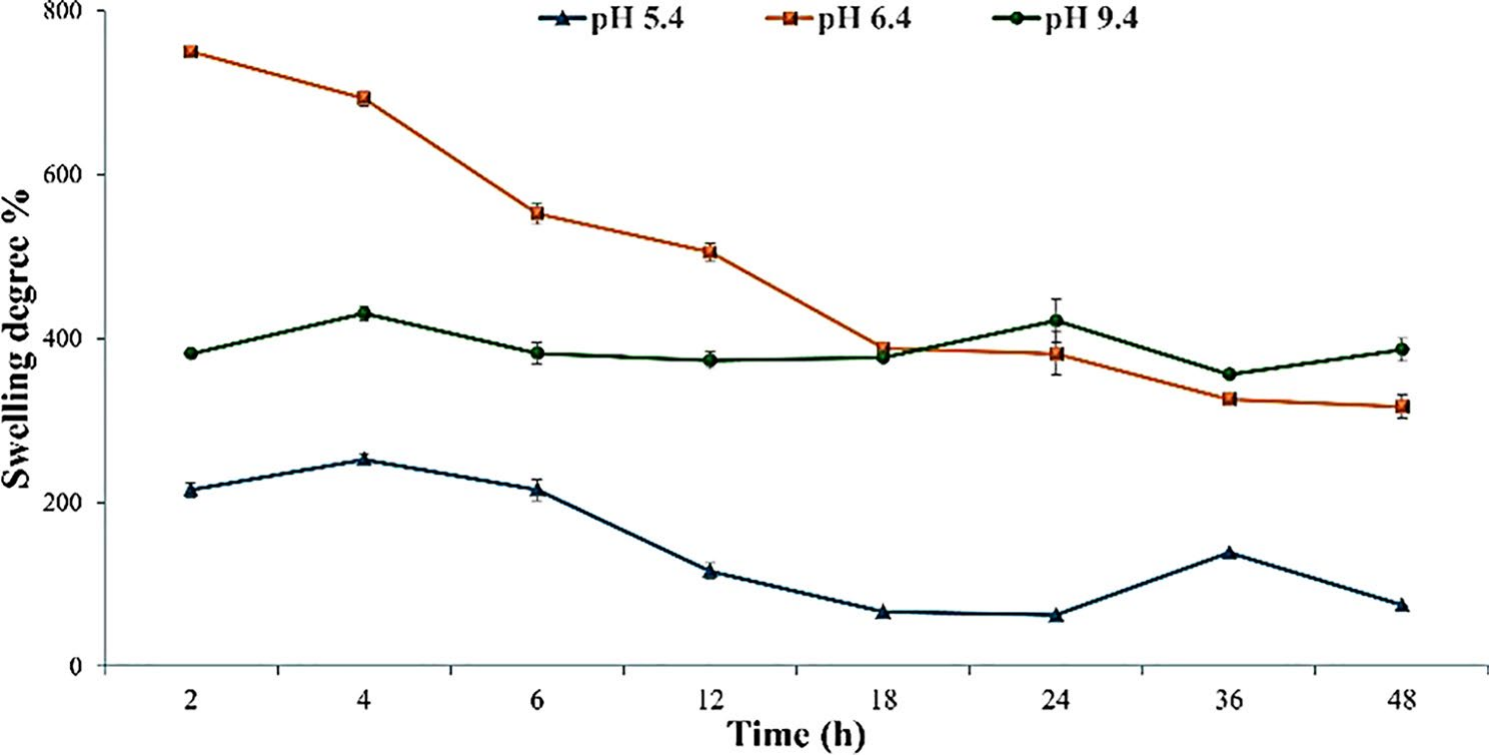
Swelling degree of Chitosan/PVA hydrogel containing ranitidine at different pH of bicarbonate buffer solution. Values are expressed as the Mean of three replicate determinations ± SEM

Figure 5 shows the swelling behavior of chitosan/PVA hydrogels at different pH values over 24 h. After 4 h, all samples exhibited noticeable swelling, with swelling ratios of approximately 252.06 ± 6.16 at pH 5.4, 692.96 ± 22.18 at pH 6.4, and 430.55 ± 9.32 at pH 9.4. The swelling ratio increased significantly with increasing pH, indicating the pH-responsive nature of the hydrogel. During the first 12 h, the hydrogel immersed in bicarbonate buffer at pH 6.4 demonstrated a higher swelling degree (505.18 ± 17.23) compared to pH 5.4 (116.10 ± 9.45) and pH 9.4 (373.26 ± 10.37), suggesting optimal hydration under mildly acidic conditions during the early phase. However, after 24 h, the maximum swelling ratio was observed at pH 9.4 (421.66 ± 26.11), indicating a delayed but enhanced swelling in alkaline environments.

#### Degradation behavior

**Fig. 6:**
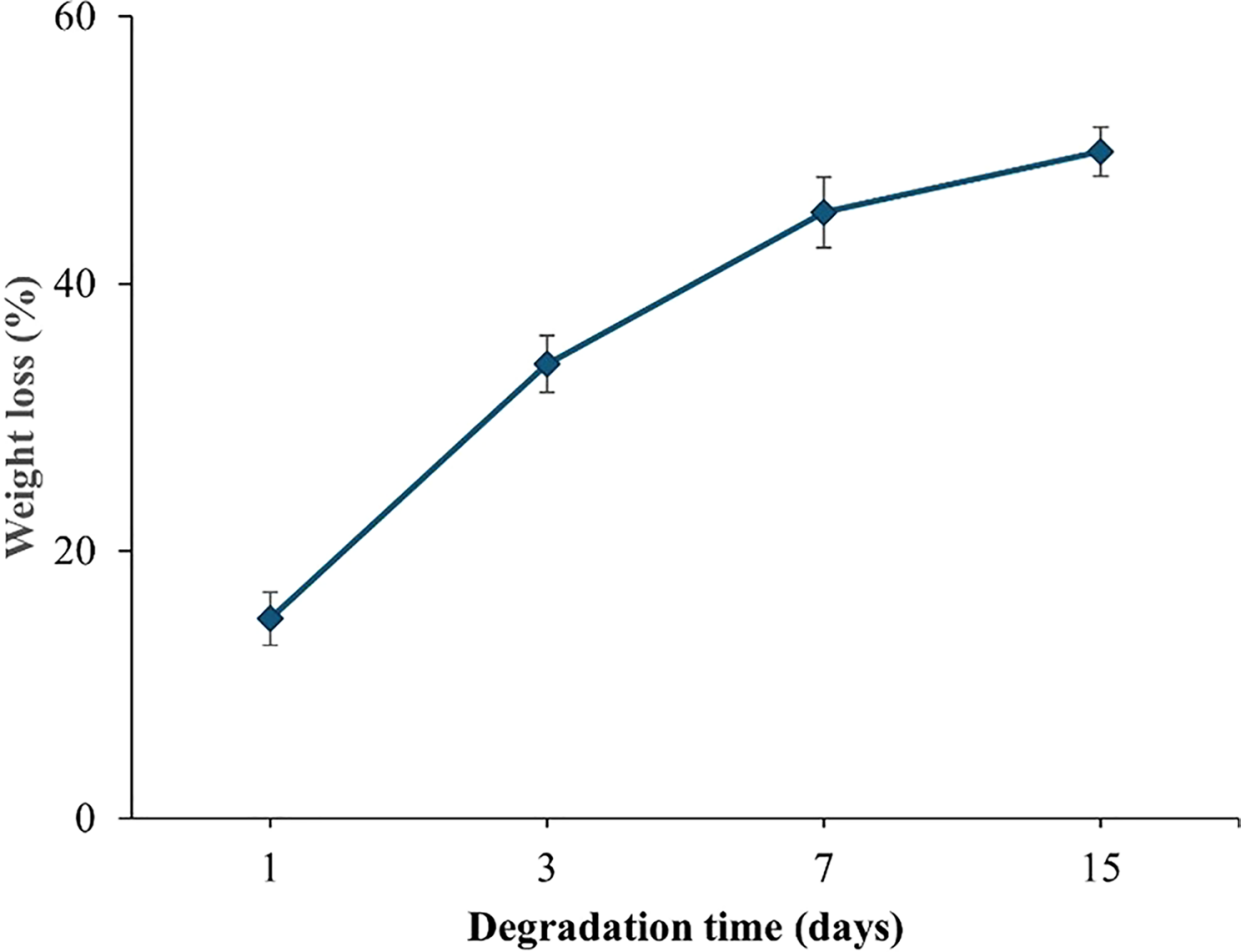
Gradual weight loss (%) of Chitosan/PVA hydrogel containing ranitidine in PBS at 37 ^◦^C. Values are expressed as the mean of three replicate determinations ± SEM

Figure 6 shows the degradation profile of the chitosan/PVA hydrogel loaded with ranitidine over 15 days, measured by weight loss in PBS (pH 1.5) at 37 °C. On day 1, the hydrogel lost 14.94 ± 1.97% of its weight. A slow degradation rate was seen between days 1 and 3, followed by a significant increase from day 7 onward. By day 15, the hydrogel lost about 49.88 ± 1.83% of its original weight, showing a steady and ongoing degradation over time.

#### Moisture retention capacity

**Fig. 7:**
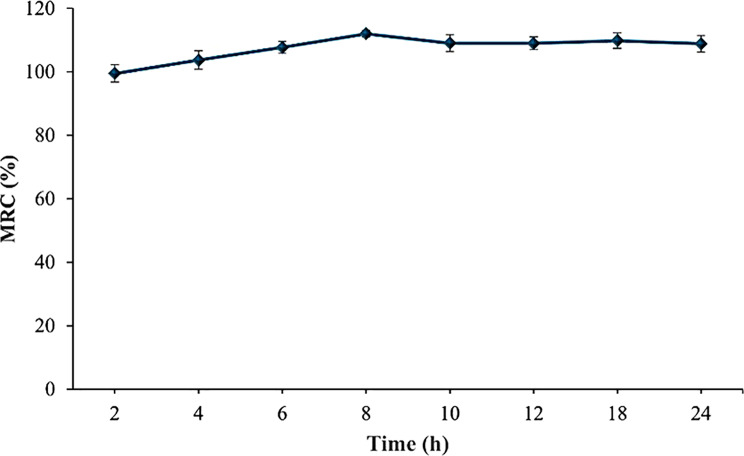
Moisture retention capability at different time intervals of Chitosan/PVA hydrogel containing ranitidine. Values are expressed as means of three replicate determinations ± SEM

Figure 7 illustrates the moisture retention capacity of the chitosan/PVA hydrogel integrated with ranitidine, which is assessed to verify its suitability for controlled drug release. After two hours, the hydrogel maintained approximately 100% moisture content, and this high retention capacity was sustained throughout a 24-hour period, indicating excellent structural stability and hydration preservation. These findings support the hydrogel’s potential for prolonged drug delivery applications.

#### In vitro Ranitidine release

**Fig. 8:**
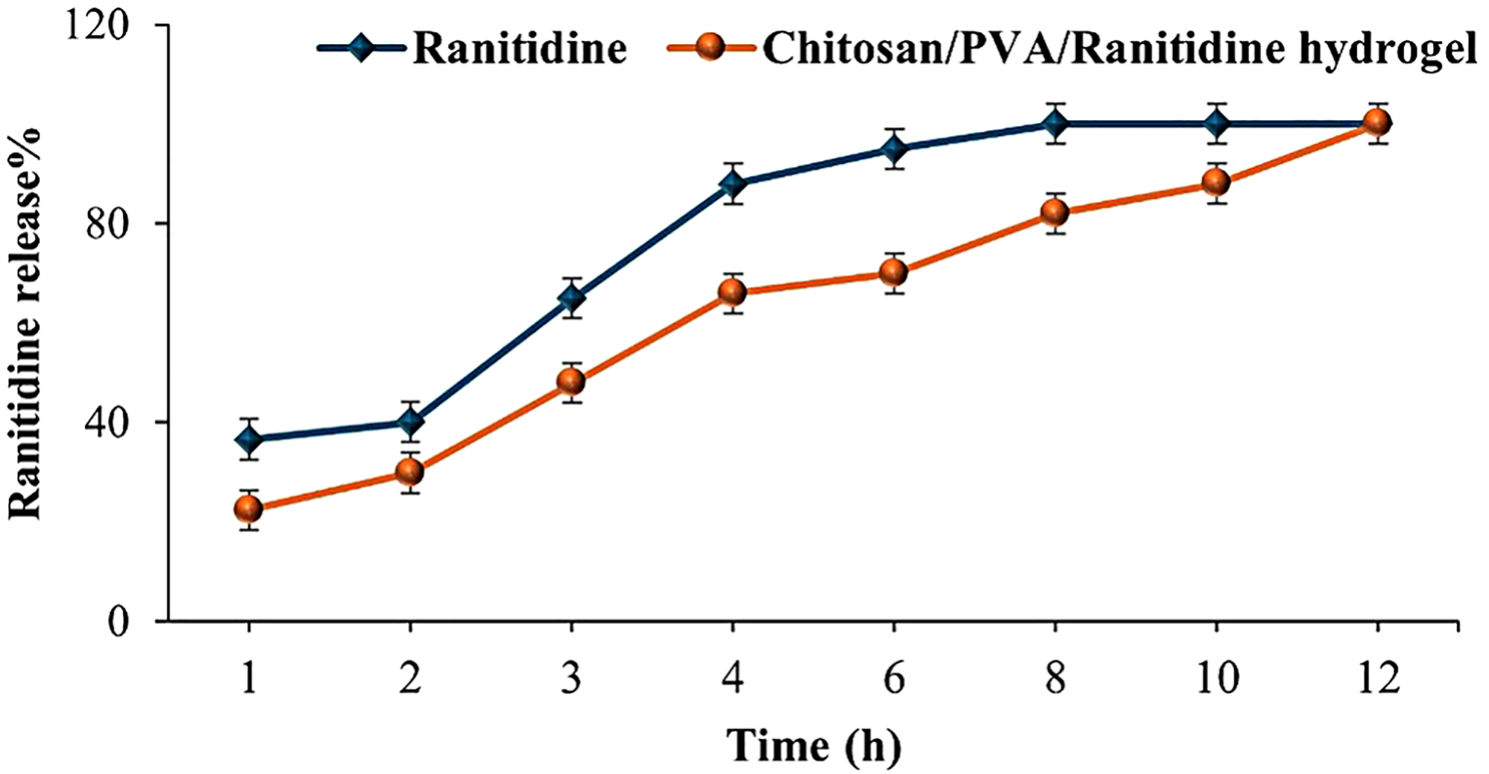
The in vitro release behavior of Ranitidine and Chitosan/PVA/Ranitidine hydrogel. Values are expressed as the means of three replicate determinations ± SEM

Figure 8 compares the release profiles of free ranitidine and ranitidine encapsulated within the chitosan/PVA hydrogel. Free ranitidine exhibited a rapid release, with approximately 65.00 ± 4.04% diffused within 3 h and 95.00 ± 4.03% released by 6 h, indicating an immediate release pattern. In contrast, the chitosan/PVA/ranitidine hydrogel demonstrated a controlled release profile, with an initial burst release of about 30.00 **±** 4.08% in the first 2 h, followed by a sustained release reaching 88 **±** 4.03% over 10 h. These results highlight the hydrogel’s potential as a sustained drug delivery system, effectively moderating the release rate of ranitidine over time.

#### Stability studies of Chitosan/PVA/Ranitidine hydrogel

##### Thermal stability

**Fig. 9:**
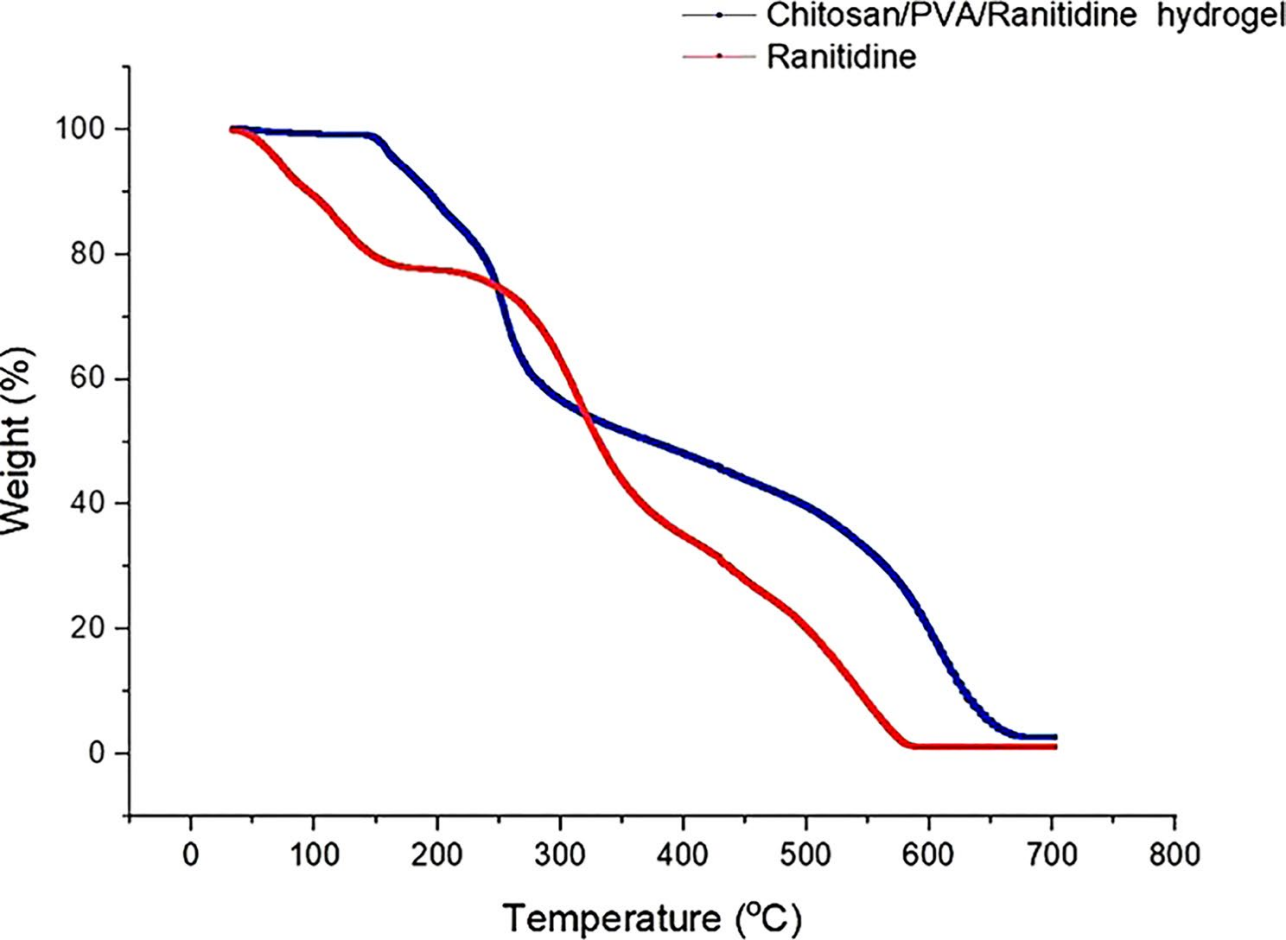
Thermal stability of Ranitidine and Chitosan/PVA/Ranitidine hydrogel using Thermal gravimetric analysis (TGA)

Figure 9 shows the thermal stability of the chitosan/PVA/ranitidine hydrogel compared to pure ranitidine, as evaluated by thermogravimetric analysis (TGA). The hydrogel demonstrated better thermal stability, showing minimal weight loss over a wide temperature range. An initial weight loss of 12.75% occurred around 100 °C, due to the evaporation of free and bound water. The hydrogel stayed thermally stable up to 200 °C, after which a significant weight decrease was seen between 200 °C and 500 °C. By 700 °C, the total weight loss was 85.87%. These findings confirm the improved thermal resistance of the chitosan/PVA matrix, confirming its potential for use in applications that need stability at high temperatures.

##### Physical stability

Table 1 shows the results of the physical stability study of the chitosan/PVA/ranitidine hydrogel stored at 4 °C for one month. The formulation stayed colloidally stable over time, with only minor variations in key parameters such as average hydrodynamic diameter, zeta potential, encapsulation efficiency, and drug-loading content. These slight changes confirm that the hydrogel keeps its physicochemical integrity during storage, supporting its potential for long-term pharmaceutical use.

**Table 1:** Storage stability of Chitosan/PVA/Ranitidine hydrogel at 4 °C

Physicochemical attributes	Chitosan/PVA/Ranitidine hydrogel	
	Before	After 30 days
Zeta potential (mv)	38.00 ± 1.41	37.26 ± 1.33
Mean particle size (nm)	69.00 ± 0.33	72.10 ± 1.16
Entrapment efficiency (%)	98.66 ± 1.01	97.86 ± 1.01
Drug loading content (%)	49.82 ± 1.29	49.43 ± 1.12

Values are means of three replicate determinations ± SEM

##### pH stability

**Fig. 10:**
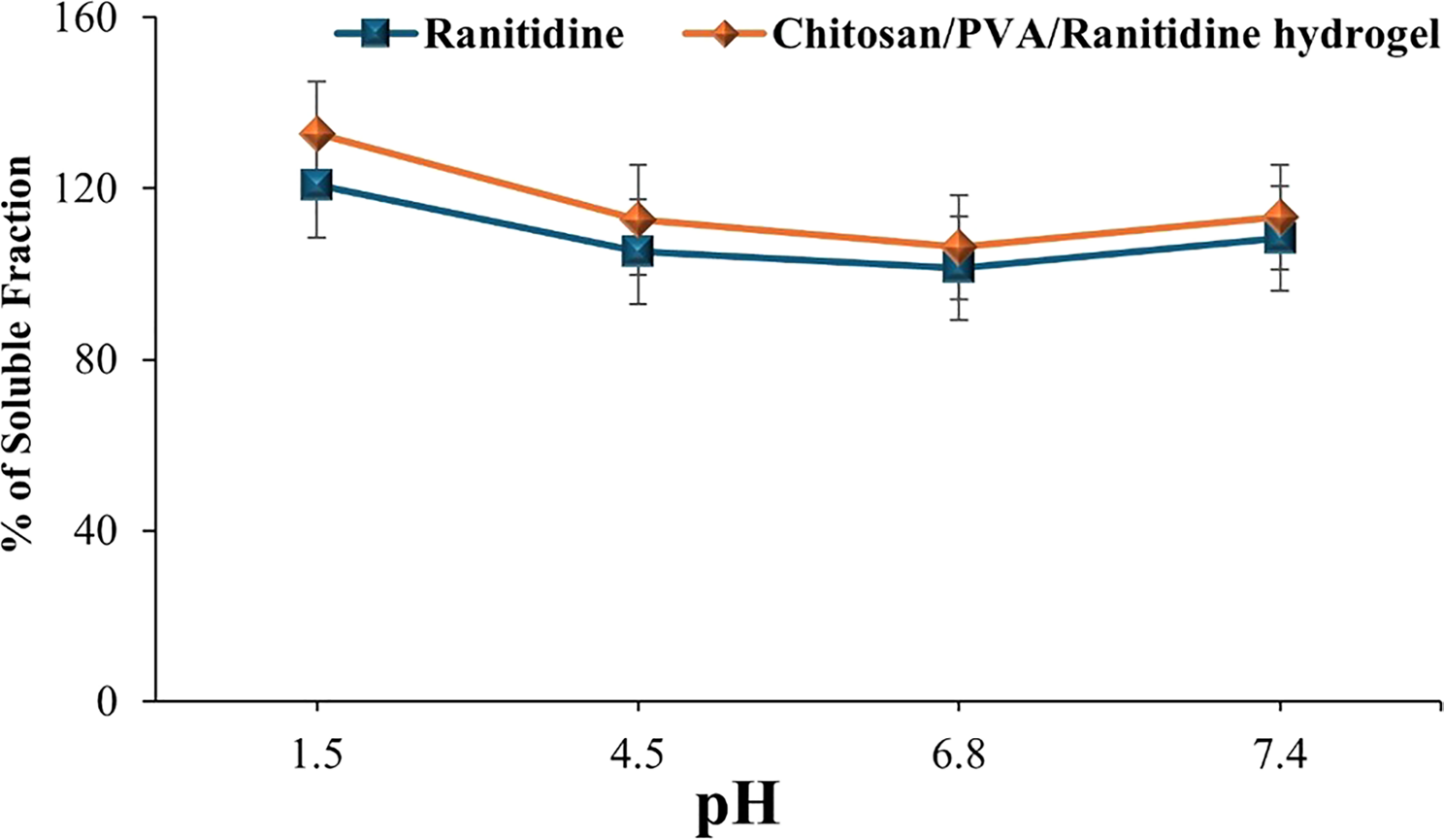
Stability of Ranitidine and Chitosan/PVA/Ranitidine hydrogel at different pH. Values are expressed as the means of three replicate determinations ± SEM

Figure 10 shows the pH-dependent stability of the chitosan/PVA/ranitidine hydrogel under simulated gastrointestinal conditions (pH 1.5, 4.5, 6.8, and 7.4). The stability was assessed by measuring the soluble fraction percentage after incubation at each pH. The hydrogel remained fairly stable in PBS at pH 1.5 and pH 7.4, showing high solubility with minimal aggregation (132.82 ± 12.10% at pH 1.5 and 113.31 ± 12.22% at pH 7.4). In comparison, free ranitidine displayed considerably lower solubility under these conditions, with soluble fractions of 120.80 ± 12.22% at pH 1.5 and 108.37 ± 12.21% at pH 7.4, indicating partial degradation and decreased stability. At pH 4.5 and pH 6.8, the hydrogel showed slightly reduced stability, with soluble fractions of 112.65 ± 12.88% and 106.26 ± 12.22%, respectively, due to mild precipitation. Nonetheless, the hydrogel still performed better than free ranitidine, which had lower soluble fractions of 105.27 ± 12.20% at pH 4.5 and 101.43 ± 12.10% at pH 6.8, suggesting a higher susceptibility to precipitation or degradation in these environments. These results indicate that the chitosan/PVA/ranitidine hydrogel has superior colloidal stability and solubility across a broad pH spectrum, especially in gastric and intestinal conditions. This supports its potential use for oral drug delivery by enhancing drug protection and stability within physiological environments.

##### Gastrointestinal stability

**Fig. 11:**
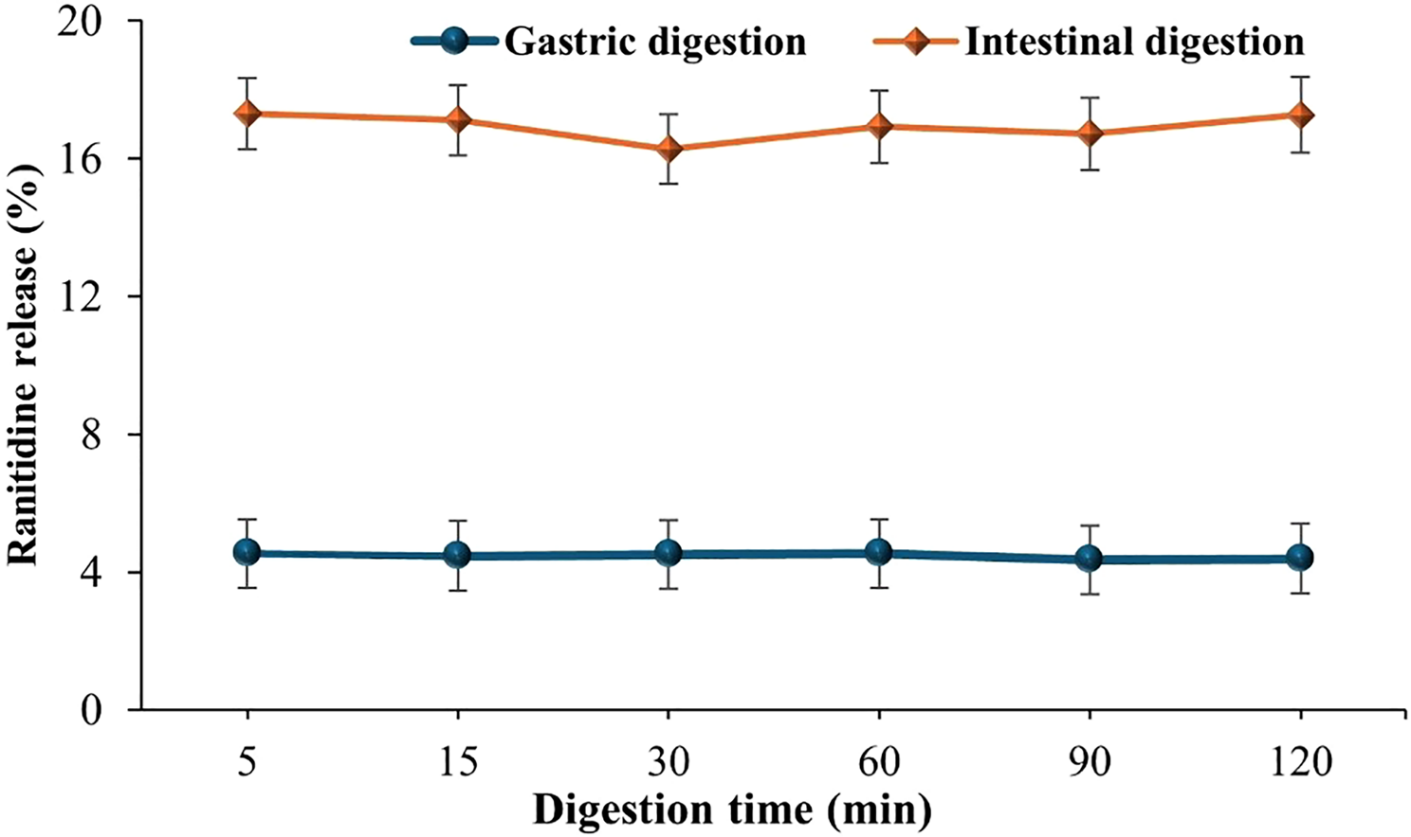
Stability of Chitosan/PVA/Ranitidine hydrogel during in vitro simulated gastrointestinal digestion. Values are expressed as the means of three replicate determinations ± SEM

During the first 30 min of simulated digestion, Ranitidine showed a faster release under intestinal conditions compared to gastric conditions, with release rates of 16.26 ± 1.01% and 4.5 ± 1.00%, respectively (Fig. 11). After 120 min, approximately 83% of the drug remained in the intestinal simulation medium, while over 95% was retained under gastric conditions. These results indicate that the Chitosan/PVA/Ranitidine hydrogel demonstrates strong structural integrity and drug retention in both gastrointestinal environments, especially in acidic gastric conditions. This stability supports the hydrogel’s suitability for oral delivery, ensuring minimal premature drug release in the stomach and allowing sustained release in the intestinal tract.

#### Cytotoxicity of Chitosan/PVA/Ranitidine hydrogel

##### Morphological changes

**Fig. 12:**
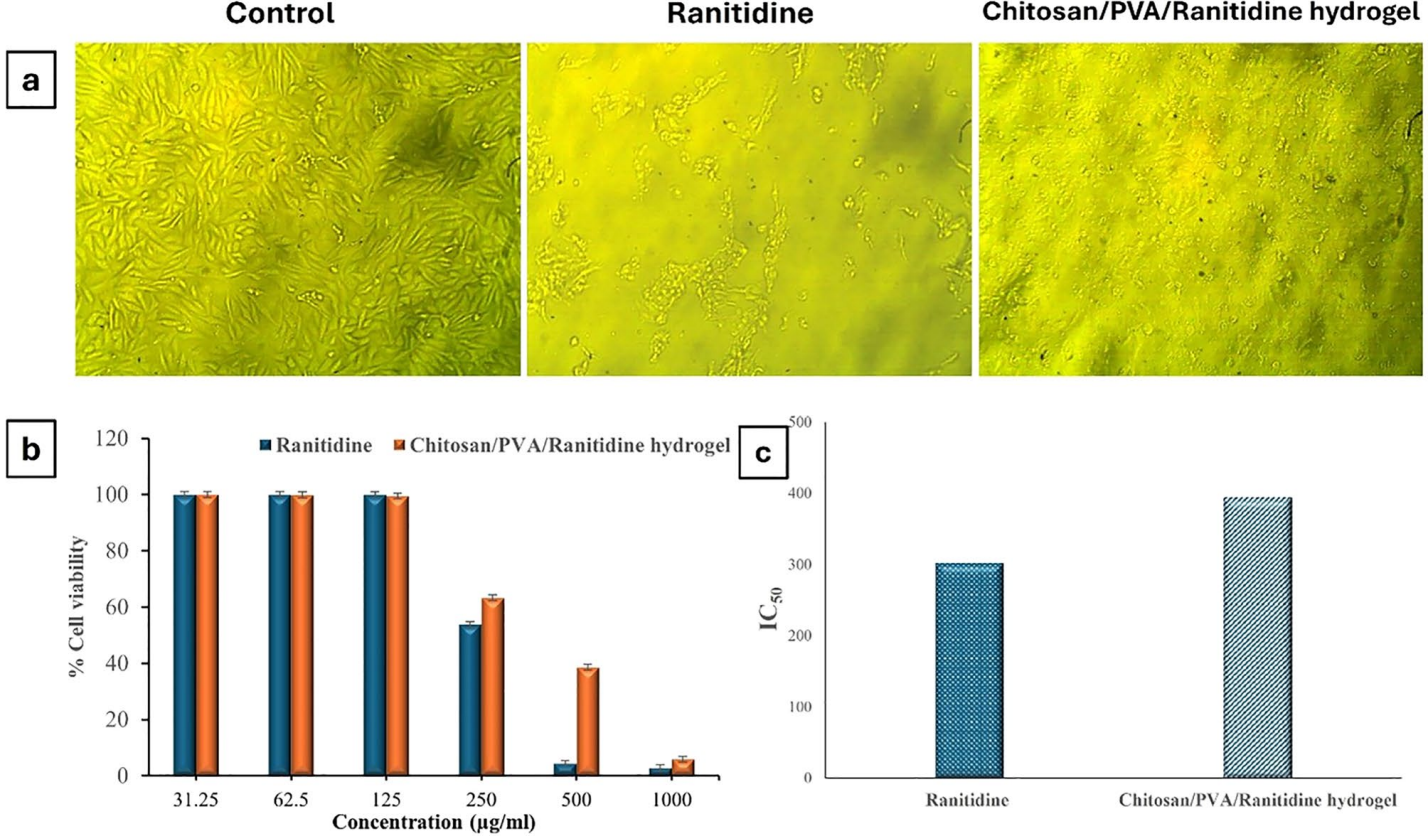
Cytotoxicity of Ranitidine and Chitosan/PVA/Ranitidine hydrogel. (**a**) Micrographs of CCL-81 cell line treated with Ranitidine and Chitosan/PVA/Ranitidine hydrogel at 500 µg/ml. (**b**) The Viability percentage of the CCL-81 cell line against ranitidine and chitosan/PVA/ranitidine hydrogel at different concentrations. (**c**) IC_50_ values of Ranitidine and Chitosan/PVA/Ranitidine hydrogel. Values are expressed as the means of three replicate determinations ± SEM

After exposure to Ranitidine at a concentration of 500 µg/mL, the CCl-81 cell line demonstrated characteristic signs of apoptosis, including significant cell shrinkage, nuclear condensation, and nuclear fragmentation compared to the control cell (Fig. 12a). Conversely, treatment with the Chitosan/PVA/Ranitidine hydrogel (500 µg/mL) resulted in fewer degenerative changes in CCL-81 when compared to untreated cells. These findings suggest that the Chitosan/PVA/Ranitidine hydrogel may exhibit lower toxicity than Ranitidine.

##### Cell viability

In Fig. 12b, the hydrogel demonstrated non-toxic properties to Vero cells (CCL-81). After 48 h of incubation, Vero cell viability following treatment with various concentrations (31.25–250 µg/ml) of hydrogel samples was over 63.29% compared to the control group. In contrast, Vero cells incubated with different concentrations ranging from 31.25 to 250 µg/ml of ranitidine showed viability of over 4.42% compared to the control group after the same incubation period (Fig. 12b). The IC_50_ of Chitosan/PVA/Ranitidine hydrogel (393.89 ± 7.78) is higher than that of Ranitidine (302.49 ± 2.83), indicating that the Chitosan/PVA/Ranitidine hydrogel is less toxic compared to Ranitidine (Fig. 12c).

#### Biological activities of Chitosan/PVA/Ranitidine hydrogel

##### Antioxidant activity

**Fig. 13:**
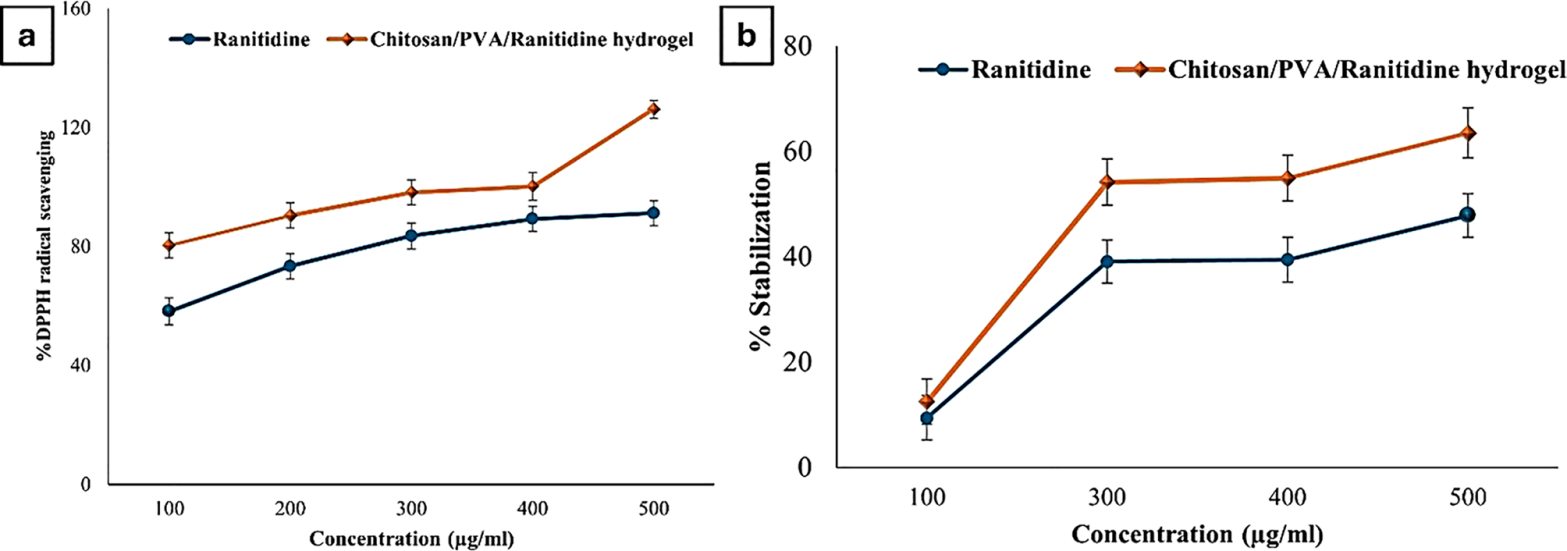
In vitro biological activities of Ranitidine and Chitosan/PVA/Ranitidine hydrogel. (**a**) DPPH radical scavenging activity of Ranitidine and chitosan/PVA/Ranitidine hydrogel. (**b**) Stabilization percentage of RBC membrane by Ranitidine and chitosan/PVA/Ranitidine hydrogel at different concentrations. Values are expressed as the means of three replicate determinations ± SEM

The experimental results show the potent antioxidant activity of the Chitosan/PVA/Ranitidine hydrogel at different concentrations (Fig. 13a). At 100 µg/ml and 500 µg/ml, free ranitidine achieved scavenging activities of 58.09 ± 4.54% and 91.17 ± 4.17%, respectively. In comparison, the hydrogel formulation demonstrated significantly higher scavenging effects, reaching 80.32 ± 4.20% at 100 µg/ml and 126.14 ± 3.10% at 500 µg/ml.

##### Anti-inflammatory activity

The anti-inflammatory efficacy of Ranitidine and the Chitosan/PVA/Ranitidine hydrogel was evaluated at various concentrations by assessing their capacity to stabilize the red blood cell (RBC) membrane (Fig. 13b). At a concentration of 100 µg/ml, Ranitidine demonstrated RBC membrane stabilization percentage of 9.41 ± 0.21%, whereas the Chitosan/PVA/Ranitidine hydrogel exhibited 12.46 ± 0.29%. The findings further reveal a significant increase in membrane stabilization percentages at concentrations ranging from 300 to 500 µg/ml, indicating enhanced anti-inflammatory activity of the hydrogel formulation. The maximum membrane stabilization observed was 47.83 ± 4.17% for Ranitidine and 63.49 ± 4.77% for the hydrogel formulation at 500 µg/ml.

### In vivo studies

#### Analgesic potency of Chitosan/PVA/Ranitidine hydrogel

##### Peripheral analgesic potency

Table 2 shows that the Chitosan/PVA/Ranitidine hydrogel and Ranitidine exhibit a peripheral analgesic effect comparable to diclofenac, indicating they inhibit the writhing response in mice. Mice treated with Chitosan/PVA/Ranitidine hydrogel and Ranitidine (8 mg/kg body weight) or standard sodium diclofenac (50 mg/kg body weight) demonstrated a significant (*P* < 0.05) reduction in the number of writhes compared to control mice. Also, the Chitosan/PVA/Ranitidine hydrogel treatment showed more pain-relieving effects in the peripheral, reaching a maximum inhibition of 59.91 ± 2.66% in the writhing reflex. This was similar to standard sodium diclofenac, which achieved an inhibition of 49.58 ± 5.52%.

**Table 2:** Peripheral analgesic potency of ranitidine and Chitosan/PVA/Ranitidine hydrogel using acetic acid-induced writhing

Experimental groups	Number of Writhing	%Inhibition
Control	48.40 ± 2.65^b^	--------
Ranitidine	24.00 ± 2.32^a^	59.41 ± 4.80 ^a^
Chitosan/PVA/Ranitidine hydrogel	19.40 ± 1.28^a^	59.91 ± 2.66 ^a^
Sodium diclofenac	24.40 ± 2.67^a^	49.58 ± 5.52 ^a^

Values are presented as means ± standard errors (*n* = 5)

Values with different superscript letters significantly differ (*P* < 0.05)

##### Central analgesic potency

As shown in Table 3, the Chitosan/PVA/Ranitidine hydrogel exhibited a significantly longer latency time across all observation periods compared to the control group, indicating a sustained central analgesic effect. While free ranitidine elicited a mild analgesic response, this became apparent at 60 min post-administration. Sodium diclofenac exhibited a rapid onset within 30 min, followed by a noticeable decline at 60 min. Notably, both ranitidine and the hydrogel formulation achieved significantly higher inhibition rates of 57.97 ± 7.30% and 67.40 ± 4.80%, respectively, exceeding the performance of sodium diclofenac. These results emphasize the hydrogel system’s prolonged and enhanced central analgesic effectiveness.

**Table 3:** Central analgesic effect of ranitidine and Chitosan/PVA/Ranitidine hydrogel using hot plate assay

Experimental groups	Latency time (sec)			%Inhibition
	30 min	60 min	90 min	
Control	13.00 ± 1.50^a^	2.20 ± 0.80^a^	1.40 ± 0.20^a^	-----
Ranitidine	13.80 ± 1.20^a^	3.60 ± 0.40^b^	3.00 ± 0.60^b^	57.97 ± 7.30^b^
Chitosan/PVA/Ranitidine hydrogel	45.00 ± 5.40^b^	4.40 ± 0.40^b^	3.40 ± 0.50^b^	67.40 ± 4.80^b^
Sodium diclofenac	36.60 ± 5.90^b^	2.40 ± 0.20^a^	2.80 ± 0.20^b^	10.56 ± 0.40^a^

Values are presented as means ± standard errors (*n* = 5)

Values with different superscript letters significantly differ (*P* < 0.05)

#### Antiulcerogenic potency of Chitosan/PVA/Ranitidine hydrogel

##### Locomotion and anxiety behaviors

A statistically significant (*P* < 0.05) decline was noted in the crossing frequency, center entry, and rearing behaviors of the ulcerated rats in comparison to the control group (Table 4). Furthermore, the ulcerated group displayed notably diminished activity levels, indicating pain and distress. Conversely, the treated groups exhibited significantly increased movement, particularly the Chitosan/PVA/Ranitidine hydrogel-treated group, which demonstrated the highest levels of locomotion. Additionally, the ulcerated rats treated with Chitosan/PVA/Ranitidine displayed greater activity compared to those treated with Ranitidine alone, illustrating the efficacy of the Chitosan/PVA/Ranitidine hydrogel in ulcer treatment.

**Table 4:** Locomotion and anxiety behaviors of different treatment groups

Experimental groups	Crossing number	Rearing	Center entry
Control	45.00 ± 5.99^b^	8.60 ± 0.49^b^	4.50 ± 0.42^c^
Ulcerated group	9.00 ± 1.59^a^	3.83 ± 0.60^a^	1.30 ± 0.41^a^
Ulcer + Ranitidine	47.00 ± 5.66^b^	9.50 ± 0.42^b^	1.30 ± 0.42^a^
Ulcer + Chitosan/PVA Hydrogel	61.16 ± 10.19^b^	11.60 ± 1.05^b^	1.80 ± 0.30^a^
Ulcer + Chitosan/PVA/Ranitidine Hydrogel	56.16 ± 7.56^b^	19.00 ± 3.22^c^	3.16 ± 0.47^b^

Values are presented as means ± standard errors (*n* = 6)

Values with different superscript letters significantly differ (*P* < 0.05)

##### Gross morphological change of gastric mucosa

**Fig. 14:**
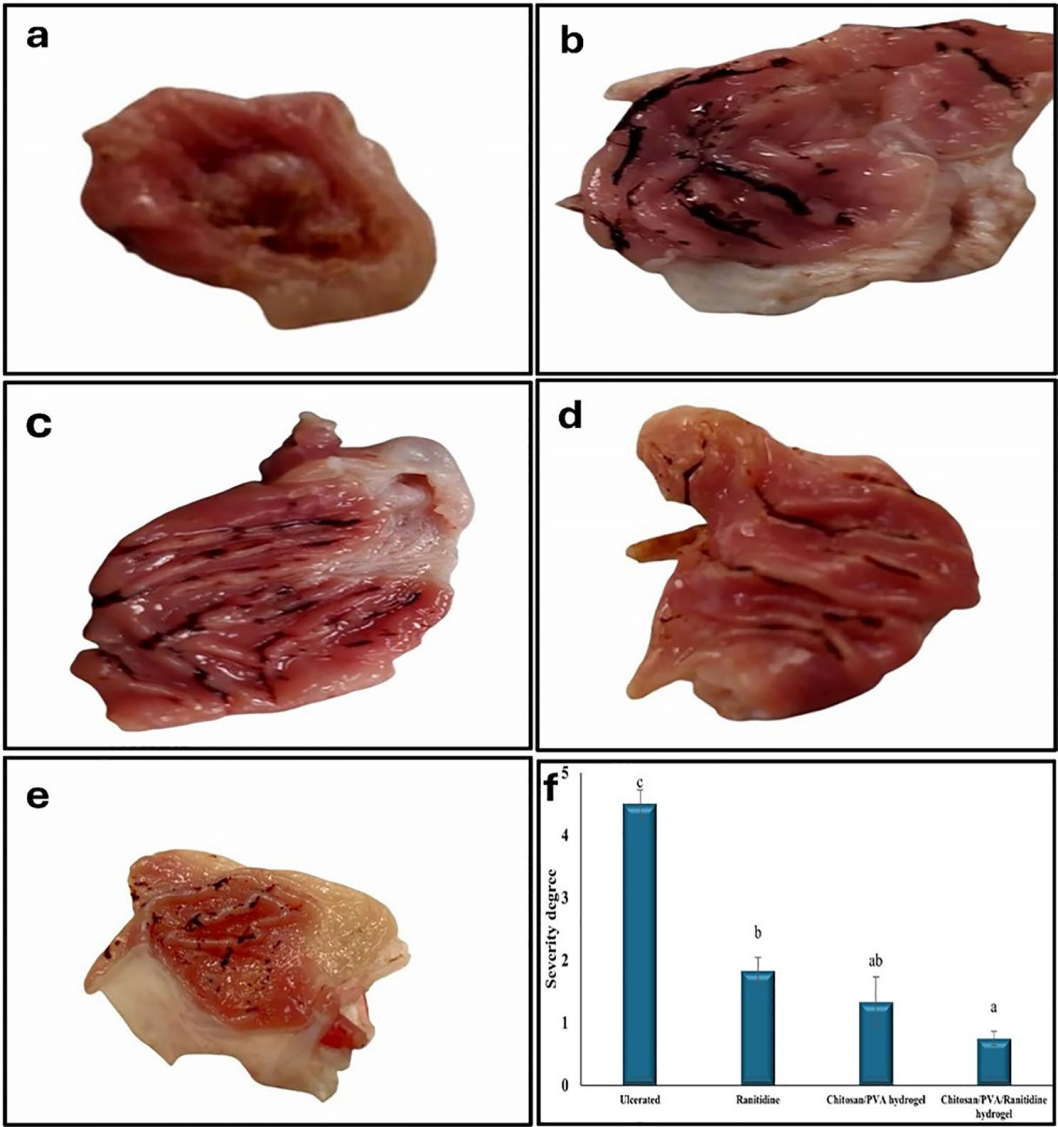
Gross morphological change of gastric mucosa. (**a**) The normal control group had a normal gastric mucosa with no signs of hemorrhagic lesions or ulceration. (**b**) Ulcerated rats displayed extensive and severe hemorrhagic gastric mucosal lesions. (**c**) Ulcerated rats treated with Ranitidine showed fewer hemorrhagic lesions of the gastric mucosa. (**d**) Ulcerated rats treated with Chitosan/PVA hydrogel showed a slight recovery. (**e**) Ulcerated rats treated with Chitosan/PVA/Ranitidine hydrogel showed a marked recovery with no hemorrhagic bands or injuries. (**f**) Gastric severity score of different ulcerated groups. Values are expressed as mean ± SEM (*n* = 6). Values with different superscript letters are significantly different (*P* < 0.05)

Figure 14a demonstrates that the control group of rats exhibited a standard gastric mucosa architecture, devoid of hemorrhagic lesions or ulceration. In contrast, the ulcerated rats demonstrated severe damage and hemorrhagic the gastric mucosa (Fig. 14b). Notably, the severity score of the ulcerated group markedly increased to 4.50 ± 0.22 when compared to the control group (Fig. 14f). Using ranitidine led to a modest improvement in gastric ulcers, as shown by reduced bleeding and hyperemia, and a significant drop in the severity score of the ulcer to 1.83 ± 0.21 compared to untreated ulcerated rats. (Fig. 14c). Notably, treatment with the Chitosan/PVA/Ranitidine hydrogel resulted in significant recovery from gastric ulcers, as shown by the lack of hemorrhagic bands or injuries, and a notable decrease in the ulcer’s severity score to 0.75 ± 0.12 compared to untreated ulcerated rats (Fig. 14e&f).

##### The ulcer index and preventive index

The statistical analysis indicates the presence of significant variances among the treatment groups. The ulcerated group showed the highest ulcer index (286.6 ± 8.82), followed by the Ranitidine group (218.80 ± 8.50), the free Chitosan/PVA hydrogel group (102.50 ± 11.50), and the Chitosan/PVA/Ranitidine hydrogel group (97.20 ± 10.05), as shown in Table 5. The Chitosan/PVA/Ranitidine hydrogel-treated group showed the highest preventive index at 66.08%, highlighting its effectiveness in stopping ulcer formation. The free Chitosan/PVA hydrogel-treated group also had a significant preventive effect at 53.13%, while the Ranitidine-treated group had the lowest preventive index at 23.64%.

**Table 5:** The ulcer index and preventive index for different treatments groups

Experimental groups	Ulcer index	Preventive index
Ulcerated group	286.6 ± 8.82^c^	—
Ulcer + Ranitidine	218.80 ± 8.50^b^	23.64
Ulcer + Chitosan/PVA hydrogel	102.50 ± 11.50^a^	53.13
Ulcer + Chitosan/PVA/Ranitidine hydrogel	97.20 ± 10.05^a^	66.08

The ulcer index values are presented as means ± standard errors (*n* = 6)

Values with different superscript letters significantly differ (*P* < 0.05)

##### Gastric juice volume, pH, and total acidity

As shown in Table 6, combining indomethacin and cold stress resulted in a significant (*P* < 0.05) rise in gastric content volume and total acidity in rats, suggesting the development of severe gastric ulcers compared to the control group. Additionally, a significant (*P* < 0.05) drop in pH was observed after combining indomethacin and cold stress compared to the control group, indicating increased gastric acid secretion. Notably, ulcerated rats treated with Ranitidine and Chitosan/PVA/Ranitidine hydrogel showed a significant (*P* < 0.05) improvement in gastric secretion indices, demonstrating its effectiveness in promoting healing (Table 6).

**Table 6:** Gastric juice volume, pH, and total acidity for different treatment groups

Experimental groups	Volume (ml)	pH	Total acidity (meq/L)
Control	1.28 ± 0.13 ͣ	2.80 ± 0.29 ͣ	73.33 ± 4.94 ͣ
Ulcerated Group	3.36 ± 0.40 ͩ	1.57 ± 0.14ᵇ	131.67 ± 9.80ᵇ
Ulcer + Ranitidine	2.11 ± 0.15ᵇ	3.06 ± 0.28 ͣ	61.67 ± 7.49 ͣ
Ulcer + Chitosan/PVA Hydrogel	2.33 ± 0.13 ͨ	2.48 ± 0.25 ͣ	78.33 ± 7.49 ͣ
Ulcer + Chitosan/PVA/Ranitidine hydrogel	1.53 ± 0.15 ͣ	3.16 ± 0.38 ͣ	58.33 ± 6.54 ͣ

Values are presented as means ± standard errors (*n* = 6)

Values with different superscript letters significantly differ (*P* < 0.05)

##### Oxidative stress markers

Relative to the control group, the gastric ulcerated group exhibited a significant (*p* < 0.05) increase in MDA content, as shown in Table 7. This rise was reversed in the Chitosan/PVA/Ranitidine hydrogel and free Chitosan/PVA hydrogel-treated mice, suggesting the potential protective effects of these treatments against oxidative stress induced by gastric ulcers. Additionally, a significant (*p* < 0.05) decrease was noted in the levels of GSH, SOD, CAT, and GPx in the ulcerated rats compared to the control group. The Chitosan/PVA/Ranitidine hydrogel-treated rats demonstrated a marked increase in these parameters, indicating their efficacy. The Ranitidine and free Chitosan/PVA hydrogel groups also significantly enhanced these antioxidative biomarkers compared to the gastric ulcer group.

**Table 7:** Gastric oxidative/antioxidative stress markers for different treatment groups

Experimental groups	MDA (nM/g.tissue)	GSH (mM/g.tissue)	SOD (U/g.tissue)	CAT (U/min/g.tissue)	GPX (µM/g.tissue/min)
Control	0.29 ± 0.17 ͣ	23636.40 ± 439.85^a^	743.37 ± 63.65^c^	43603.24 ± 1611.31^c^	0.47 ± 0.08^c^
Ulcerated group	4.49 ± 0.57ᵇ	19259.05 ± 215.02^a^	229.16 ± 2.76^a^	18506.52 ± 57.56^a^	0.19 ± 0.03^a^
Ulcer + Ranitidine	0.31 ± 0.01^a^	29674.43 ± 2264.04^b^	392.00 ± 55.81^ab^	30018.64 ± 23.03^b^	0.35 ± 0.01^bc^
Ulcer + Chitosan/PVA hydrogel	0.25 ± 0.01^a^	23312.62 ± 2343.42^a^	627.96 ± 152.5^bc^	21562.39± 24.0^a^	0.27± 0.01^ab^
Ulcer + Chitosan/PVA/Ranitidine hydrogel	0.22 ± 0.01^a^	32434.69 ± 2402.53^b^	471.35 ± 71.58^ab^	30670.56 ± 23.010^b^	0.41 ± 0.05^bc^

Values are presented as means ± standard errors (*n* = 6)

Values with different superscript letters significantly differ (*P* < 0.05)

##### Histological analysis

**Fig. 15:**
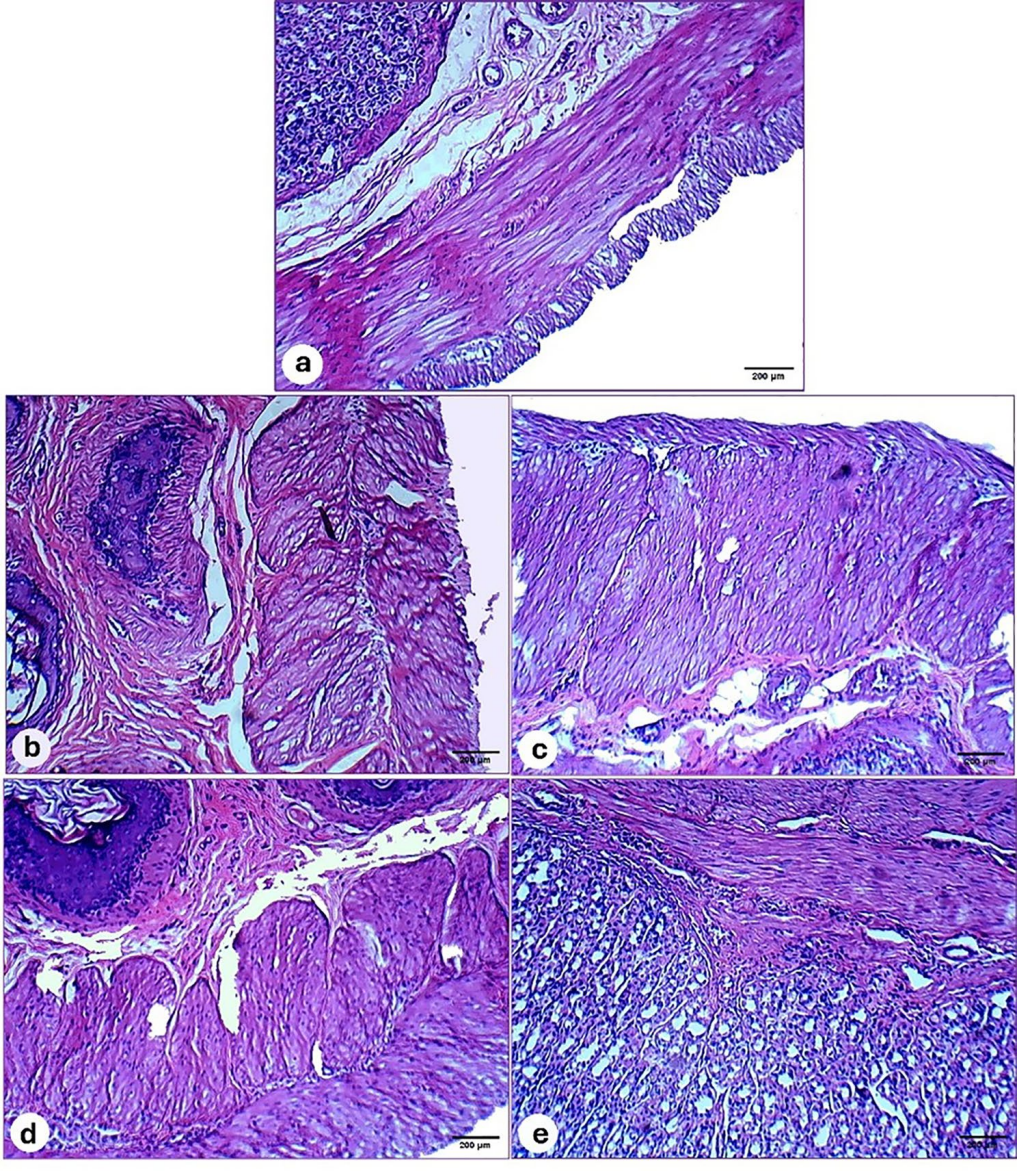
Photomicrographs of the rat’s gastric mucosa architecture of different experimental groups stained with hematoxylin and eosin. (**a**) control group showing the well-preserved normal histological structure of stomach mucosa. (**b**) ulcerated group showing severe alterations in gastric mucosa with higher power of ulcerative mucosal surface. (**c-d**) Ulcerated rats treated with ranitidine and Chitosan/PVA hydrogel showed a slight improvement in mucosa structure, confirmed by slight normalization of mucosa. (**e**) Ulcerated rats treated with chitosan/PVA/ ranitidine hydrogel showed a pronounced recovery in the gastric mucosa

Figure 15a presents the normal histology of gastric tissue within the control group, depicting a typical histological configuration of the gastric mucosa and submucosa. Conversely, the gastric architecture of untreated ulcerated rats displayed significant histopathological alterations. This was evidenced by the presence of multifocal ulcerative areas in the glandular mucosa, characterized by the desquamation of the epithelial lining, accumulation of necrotic tissue, and pronounced infiltration of inflammatory cells (Fig. 15b). In contrast, ulcerated rats subjected to treatment with either Ranitidine or Chitosan/PVA hydrogel demonstrated a modest improvement in gastric structure. This was corroborated by the observation of mild epithelial sloughing and reduced infiltration of inflammatory cells in the mucosa and submucosal layer (Fig. 15c & d). Notably, ulcerated rats treated with Chitosan/PVA/Ranitidine hydrogel significantly recovered the gastric mucosa. This was evidenced by restoring the standard histological structure of the glandular mucosa and submucosa (Fig. 15e).

##### Mucosal glycoproteins

**Fig. 16:**
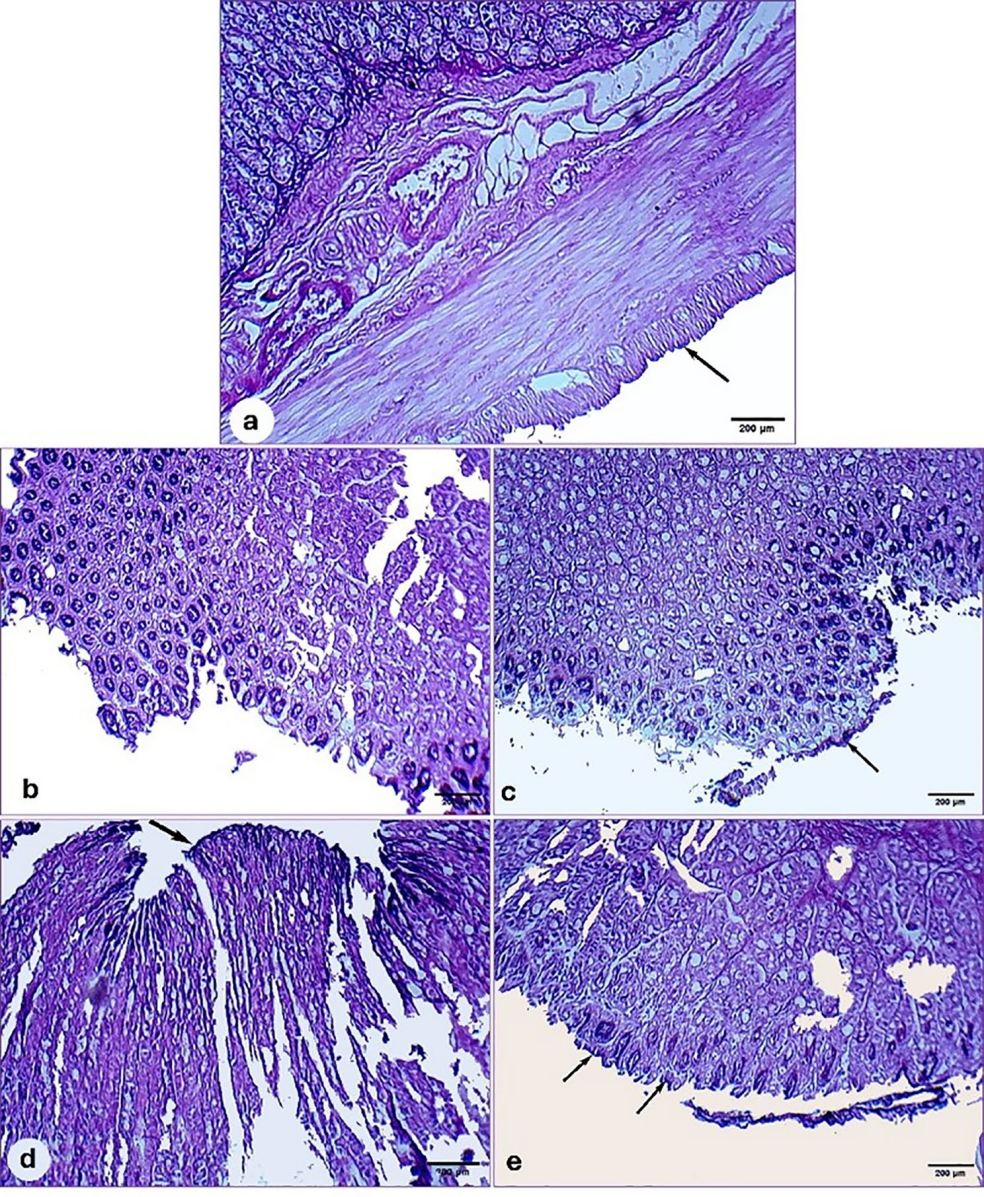
Photomicrographs of the rat’s gastric mucosa architecture of different experimental groups stained with PAS. (**a**) control group exhibiting a normal magenta coloring (black arrow) of the gastric mucus glands. (**b**) ulcerated group showing severe alterations in gastric mucosa with no PAS staining in the mucosa. (**c-d**) Ulcerated rats treated with ranitidine and free Chitosan/PVA hydrogel showed a slightly intense magenta coloring. (**e**) Ulcerated rats treated with chitosan/PVA/ ranitidine showed intense uptake of the PAS stain, indicating restoration of the mucosal barrier

Periodic Acid Schiff staining is used to detect mucosal glycoproteins. Figure 16b displayed a decreased or missing magenta color in the ulcerated group, indicating a reduction in the mucosal wall barrier compared to the control group, which exhibited normal intense magenta staining (Fig. 16a). Notably, Ranitidine and Chitosan/PVA/Ranitidine hydrogel significantly reinstated the intense magenta color, signaling the restoration of the gastric mucosal lining. It is worth noting that this effect was more pronounced for the hydrogel incorporated with Ranitidine than for Ranitidine and Chitosan/PVA hydrogel (Fig. 16c-e).

## Discussion

Ranitidine is commonly prescribed to treat peptic ulcers, heartburn, and gastroesophageal reflux disease. Prior studies show that ranitidine metabolites interact with nitrite in acidic conditions, forming NDMA, a probable human carcinogen [2]. Additionally, ranitidine has a short biological half-life of approximately 2.5 to 3 h and an absolute bioavailability of 50% [6]. As a result, a gastroretentive drug-delivery system can prolong the retention of ranitidine in the stomach, improving its local delivery and offering significant promise for therapeutic advancement. Therefore, the present study was designed to develop a chitosan-based hydrogel as a mucoadhesive/gastro-retentive delivery system for ranitidine due to its excellent biocompatibility, enhanced therapeutic effectiveness, and controlled drug release [39].

To ensure that the prepared hydrogel has the desired gastro-retentive properties, it is essential to evaluate its various physicochemical attributes comprehensively. The transmission electron microscope is crucial to assessing the morphological characteristics of a hydrogel formulation. In the current study, TEM analysis confirmed the spherical shape structure of the Chitosan/PVA/Ranitidine hydrogel. This indicates a well-organized assembly of hydrogel particles with a nano-sized dimension of 55 nm. Moreover, the dynamic light scattering (DLS) study of the Chitosan/PVA/Ranitidine hydrogel has revealed an unimodal distribution of particles, with a predominant particle size of approximately 69 nm. This uniform distribution implies a homogeneous population of particles, which is imperative for consistent drug release and predictable pharmacokinetics [40]. The research conducted by Klausner et al. [41] illustrates that particles ranging from 50 to 200 nm in size are recommended for gastroretentive formulations. These particles are optimal for augmenting mucosal adhesion and facilitating drug absorption within the gastrointestinal tract [41]. Overall, the small size of the hydrogel underscored the promise of the Chitosan/PVA hydrogel for enhancing Ranitidine’s bioavailability and therapeutic outcomes, as Irfan et al. [42] demonstrated.

Zeta potential is a crucial attribute for assessing the effectiveness of chitosan-based hydrogel.

as a gastro-retentive drug delivery system. It serves as an indicator of the hydrogel’s stability and its potential for controlled drug release. The current research illustrates that the Chitosan/PVA hydrogel provides steric stability to Ranitidine by forming a barrier between particles and the surrounding medium. This barrier generates repulsive forces between the particles, preventing them from coming into close contact. This conclusion was corroborated by a prior investigation conducted by Ghasemi et al. [43]. Therefore, the chitosan-based hydrogel protects the ranitidine and is an optimal carrier for a gastro-retentive drug delivery system. The positive zeta potential of the prepared hydrogel can be due to the presence of amino groups in chitosan, which are positively charged [44].

The chemical structure of the synthetic hydrogel was characterized, and the influence of PVA on the polymerization process was analyzed using FT-IR spectroscopy. The current FT-IR spectrum showed chitosan-specific peaks at 1636, 1577, and 897 cm^− 1^, while PVA showed OH stretch and C = O at 1709 cm^− 1^, which caused chitosan peaks to shift right due to hydrogen bonding between PVA and Chitosan’s OH and NH [45, 46]. Ranitidine samples displayed peaks at 1043 cm^− 1^ in crystalline form and bands at 1619 cm^− 1^ for the C = N of the nitronic acid, 1225 and 1380 cm^− 1^ for the nitro group, and 3258 cm^− 1^ for the dimethylamine group [47]. The FT-IR spectra of the Chitosan/PVA hydrogel containing Ranitidine exhibited three prominent peaks of Ranitidine, suggesting that the drug and polymers are compatible, as observed previously [48].

The crystal structure of a gastro-retentive drug delivery system plays a vital role in modulating the dissolution kinetics and, consequently, the bioavailability of the entrapped drug. X-ray diffraction (XRD) analysis confirmed that ranitidine exhibits a crystalline structure, which, upon incorporation into the chitosan/PVA hydrogel matrix, became molecularly dispersed within the polymer network. This distribution contributes to improved drug stability and controlled release, aligning with findings by Raza et al. [48]. The prepared chitosan/PVA hydrogel also exhibited crystalline characteristics, mainly due to the innate crystallinity of chitosan [49]. Additionally, this crystalline structure is formed by intramolecular hydrogen bonding between polymer chains within the internal structure of hydrogen, as Chen et al. [50] stated. Elevated crystallinity is linked to reduced polymer chain mobility and decreased free volume within the hydrogel matrix. Such structural rigidity hampers solvent molecule penetration and lowers the rate of drug diffusion through the matrix [51]. Consequently, crystalline regions act as barriers to water ingress and drug transport, reducing the overall dissolution rate of the embedded drug [52]. Additionally, these crystalline domains increase crosslink density, enhancing the hydrogel’s mechanical stability and swelling resistance under physiological conditions [53]. This structure benefits gastro-retentive systems by prolonging drug residence, enabling sustained release, and reducing dosing for ranitidine, which has narrow absorption and pH sensitivity.

The Chitosan/PVA hydrogel demonstrated consistent encapsulation efficiency for 30 days, indicating that the hydrogel matrix maintained its structural integrity and effectively avoided substantial loss or degradation of the entrapped ranitidine. The stability facilitated the establishment of an ideal environment for drug encapsulation and retention. The hydrogel system’s polymer components are responsible for its comparatively high encapsulation efficiency and prolonged drug-loading capability [54]. The biocompatibility and mucoadhesive qualities of chitosan improve the process of drug encapsulation and result in extended drug release [55]. In addition, the polymer structure of PVA had a role in creating a durable framework that efficiently captured drug molecules [56]. Additionally, these remarkable findings are reinforced by the small size of the hydrogel system, which provides a substantial surface area to encapsulate ranitidine, as demonstrated by Thang et al. [54].

Understanding the swelling behavior is essential for comprehending the drug delivery mechanism. A higher swelling results from a greater uptake of physiological fluids, leading to increased wetting and penetration into the hydrogel matrices. This, in turn, enhances the diffusion of drugs [57]. The present work illustrated that the pH level significantly influences the swelling characteristics of the hydrogel, and the extent of swelling intensifies as the pH level rises. The hydrogel exhibited more significant swelling over the initial 12-hour period when exposed to a bicarbonate buffer solution with a pH of 6.4. According to Timur & Paşa [58] the prepared hydrogel contains OH and unreacted NH2 groups, which facilitate the swelling process by hydrogen bonding with water. The protonation of these functional groups reduces hydrogen bonding and swelling in an acidic environment compared to a neutral environment. In addition, Rahmatpour et al. [59] hypothesized that the increased swelling rate of the chitosan/PVA/Ranitidine hydrogel could be due to its porous three-dimensional structure and abundant hydrophilic groups on the chitosan and PVA polymer chains.

Recognizing degradation is critical when utilizing biomaterials for medical applications. The duration they can remain in the body directly impacts their ability to fulfil a specific application effectively [60]. The intrinsic flexibility of polymeric materials endows them with the capacity to modulate drug release and mitigate associated adverse effects. Following a 15-day evaluation period, it was observed that the Chitosan/PVA hydrogel containing ranitidine had experienced a mere 49.8% reduction in its initial weight. This outcome suggests that the hydrogels maintained structural integrity using optimized degradation, facilitating controlled and sustained drug release. These findings corroborate previous research [61].

The research investigated the potential of the hydrogel system for controlled ranitidine release, considering its moisture retention capacity [62]. Based on the current findings, it is evident that the hydrogel exhibits a moisture retention capacity of approximately 100% over 24 h. This denotes the hydrogel’s capability to effectively absorb and retain a substantial amount of water over an extended duration. The exceptional water retention is attributed to the specialized cross-linked structure and abundant hydrophilic groups on the molecular chains [50]. The observed findings suggest a gradual release of Ranitidine from the hydrogel over an extended period, signifying significant advancements in controlled release. The drug release profile of Chitosan/PVA/Ranitidine hydrogel confirms this, showing a slow-release pattern with Ranitidine being steadily released over time. Only a small amount of Ranitidine was released after 3 h, indicating the potential for sustained release. By the 12-hour mark, about 80% of the drug had been released, showing that the hydrogel can control the release of the drug over an extended period. In contrast, the free ranitidine sample showed a quick increase in the percentage released over time, most released within the first 3 h. This is likely due to the presence of cross-linked networks and polymers that form solid and intact gel-like structures, leading to long-lasting therapeutic effects and reduced side effects [22].

One of the main challenges in preventing the use of ranitidine in the medical industry is the loss of effectiveness during product storage [63]. Thus, assessing the physicochemical properties of hydrogels during storage is essential for determining their physical stability. The physical stability results show that the hydrogel formula remains highly stable, with minimal changes in its physical and chemical attributes after being stored for 30 days at 4 °C. Furthermore, the stability of the Chitosan-based hydrogel containing Ranitidine was evaluated at different pH levels (1.6, 5.5, and 7.4) to predict its stability in the digestive tract. Ranitidine’s release rate was higher in simulated intestinal conditions compared to simulated gastric solutions. This indicates that the Chitosan/PVA/Ranitidine hydrogel maintains high stability under gastric digestion conditions. It suggests its potential as a gastro-retentive system for controlled release and stability in gastric conditions, and it can potentially have a curative effect on ulcers [64]. The hydrogel formula’s stability is due to the even distribution of charge density on its surface, achieving thermodynamic stability, as evidenced by its relatively high zeta potential. Additionally, it is essential to note that chitosan demonstrates high solubility in acidic conditions, potentially contributing to the gastric stability of the prepared hydrogel [65]. Rodríguez-Rodríguez et al. [66] revealed that the hydrogel’s encapsulation efficiency and porous structure provided environmental protection and allowed for more targeted, controlled drug release. This led to a significant increase in the hydrogel formula’s ability to stabilize the drug compared to free Ranitidine. This indicates an effective increase in drug efficacy.

The current study has confirmed the biological activity of the hydrogel system, demonstrating that both Ranitidine and Chitosan/PVA/Ranitidine hydrogel possess DPPH free radical scavenging activity. These findings are consistent with previous studies [67, 68]. Ranitidine has been reported to exhibit scavenging activity due to the presence of a furan ring in its structure [69]. Also, the current analysis has shown that Ranitidine and its hydrogel formulation exhibit notable anti-inflammatory properties by stabilizing the RBC membrane, which serves as a model of the lysosomal membrane. The results may support previous studies, suggesting that Ranitidine treatment may reduce the release of lysosomal enzymes and could be linked to intracellular OH^−^ scavenging [69]. The enhanced biological activities of ranitidine during hydrogel encapsulation may be due to the unique physicochemical characteristics of the hydrogel structure and its bioactivity, as previously demonstrated [70]. The enhanced biological activity of the ranitidine hydrogel formula may be ascribed to the incorporation of chitosan, renowned for its antioxidative and anti-inflammatory attributes [71]. Consequently, the synergistic action of chitosan and ranitidine has engendered an overall more potent antioxidative and anti-inflammatory impact within the hydrogels. Furthermore, the hydrogel containing Ranitidine showed higher cell viability and metabolic activity compared to Ranitidine and exhibited reduced toxicity in the MTT assay. The findings indicate that chitosan hydrogel may enhance cell proliferation due to its excellent cytocompatibility [72].

The enhanced central and peripheral pain-relief effects observed after the Chitosan/PVA/Ranitidine hydrogel treatment are due to multiple interconnected mechanisms linked to its physicochemical characteristics and drug release behavior [73]. In peripheral analgesia, the hydrogel’s sustained-release profile ensures prolonged exposure of ranitidine at the site of inflammation, enabling continuous inhibition of key pain mediators such as prostaglandins and bradykinin [74, 75]. The bioadhesive nature of chitosan enhances local retention and interaction with inflamed tissues, improving drug residence time and tissue penetration [76]. Moreover, chitosan possesses intrinsic anti-inflammatory and analgesic properties that may synergize with ranitidine’s pharmacological action [77]. Regarding central analgesic activity, the hydrogel protects ranitidine from rapid degradation, thus enhancing its gastrointestinal stability and systemic absorption [78]. Notably, nanoscale formulations have demonstrated improved blood brain barrier penetration through receptor-mediated transcytosis and endocytic mechanisms, enabling targeted central nervous system (CNS) delivery that may influence nociceptive signaling pathways, including those modulated by histamine H_2_ receptors and endogenous opioids [79].

Chitosan-based hydrogels incorporating ranitidine demonstrate notable structural characteristics, including porosity and swelling capacity, rendering them highly promising for ulcer treatment [80]. These hydrogels efficiently absorb exudates, maintain optimal hydration levels, facilitate gas exchange, and, when applied to ulcers, arrest bleeding and shield the ulcer from gastric fluids [81]. Collectively, these attributes address critical aspects of gastric ulcer care. The present study supports this by evaluating the prepared hydrogel formula’s anti-ulcerogenic potency.

The selected ranitidine dose for this study was based on previous experimental models showing significant anti-ulcer, antioxidant, and anti-inflammatory effects in rats. This dose has proven effective and safe for repeated use in preclinical ulcer models [36]. The same dose was used in the Chitosan/PVA/Ranitidine hydrogel formulation to directly compare the free drug and the hydrogel system, thus allowing a precise evaluation of the formulation-related improvements in pharmacological activity. The current study showed that a ranitidine hydrogel formula demonstrated gastroprotective effects and antioxidant properties in treating gastric ulcers induced by indomethacin and cold stress. Additionally, the ranitidine-containing hydrogel helped restore the stomach’s structure and relieve anxiety-like behavior and motor deficits associated with gastric ulcers. This healing potential could be credited to the formula’s antioxidant properties, as indicated in this study. Additionally, the mucoadhesive effect of the formula, attributed to the presence of chitosan, allows it to adhere to the mucosal surface through the interaction of the cationic amine groups of chitosan with anionic groups on the mucosal surface [82]. Consequently, the current investigation posits that the hydrogel formulation of ranitidine serves as a protective barrier for the stomach and demonstrates mucoadhesive properties, which are imperative for mitigating the impact of gastric acids and enzymes, recognized as predisposing factors to the development of gastric ulcers. These findings align with the previous reports [83, 84]. Furthermore, Dhaliwal et al. [85] demonstrated that the hydrophilic groups in the prepared hydrogel, namely hydroxyl and amino groups, can establish hydrogen bonds with the sialic acid found in the mucin molecule, thereby augmenting adhesion. Moreover, the observed increase in gastric pH levels after ranitidine hydrogel treatment is attributed to the presence of chitosan in the hydrogel formulation. A prior study demonstrated that chitosan could augment mucus contents, thereby impeding the influx of sodium and potassium ions into the lumen and pepsin secretion [86]. Additionally, increasing mucus content may help prevent ulcers by aiding the diffusion of hydrogen ions, strengthening acid buffering, and reducing stomach wall friction through peristalsis [87].

Gastric ulcers primarily arise from reactive oxygen species (ROS) and oxidative stress, causing cellular damage and breakdown of the mucosal lining [88]. In this study, Treatment with a hydrogel formulation of ranitidine reduces oxidative stress, as evidenced by a significant decrease in gastric malondialdehyde (MDA) levels and a concurrent increase in glutathione (GSH) content. In addition, crucial internal antioxidant enzymes, particularly SOD, CAT, and GPx, play a vital role in preventing the development of gastric ulcers [89]. These enzymes play essential roles in neutralizing ROS and protecting the gastric mucosa from oxidative injury [90], markedly increased activity following hydrogel treatment. These findings suggest that the hydrogel formulation not only preserves ranitidine’s therapeutic action but also enhances its antioxidant potential. This enhancement may be attributed to two synergistic factors: first, the controlled and sustained release of ranitidine from the hydrogel matrix, which could prolong the residence time of ranitidine in the gastrointestinal tract and enables ongoing ROS scavenging [91]; second, the inherent antioxidant properties of chitosan, recognized for its ability to scavenge free radicals and reduce inflammation [92]. These factors enhance the oxidative defense system and aid mucosal protection and healing.

A histological study supports the effectiveness of the ranitidine hydrogel formula in treating ulcers. The study revealed that the ranitidine hydrogel aided in restoring the stomach’s standard structure by reducing inflammation, promoting the formation of granulation tissue, and facilitating the rapid proliferation of epithelial cells, endothelial cells, and fibroblasts. In comparison, the ulcerated group exhibited higher levels of inflammation characterized by lymphoid aggregates and infiltration resulting from the ulceration. Additionally, the Chitosan/PVA/Ranitidine hydrogel-treated group showed the presence of epithelial cells and mucus cells, indicating tissue stability. The presence of epithelial cells in the Chitosan/PVA/Ranitidine hydrogel-treated group is due to its tissue generation ability for tissue healing [46]. The ranitidine hydrogel formula exhibited normal mucus cells, revealing its ability to maintain intact tissue. Based on the findings, it is clear that the hydrogel formula of ranitidine is more effective in healing ulcers than treating gastric ulcers with free ranitidine. This could be due to the controlled release of the drug and the maintenance of an optimal medication concentration over a longer period, as Heikal et al. [93] stated.

The therapeutic effects observed with the Chitosan/PVA/Ranitidine hydrogel may not solely be attributable to ranitidine; rather, they may also arise from the inherent bioactivity of the polymeric matrix, particularly chitosan. It is known for its wide range of biomedical applications. These include anti-ulcer, antioxidant, anti-inflammatory, and mucoadhesive properties [94]. Additionally, the positive charge of chitosan in acidic environments enables it to adhere to the negatively charged gastric mucosa, forming a protective barrier that reduces mucosal irritation and promotes local healing [95]. Additionally, chitosan has free radical scavenging ability and modulates inflammatory cytokine activity, aiding gastric mucosal protection and peripheral pain relief [96]. While polyvinyl alcohol (PVA) is pharmacologically inert, it creates a mechanically stable, hydrophilic matrix for sustained drug release and extended retention time at the administration site [97]. Thus, the combined use of ranitidine and the chitosan-based matrix enhances therapeutic effectiveness by enabling targeted delivery, extending bioavailability, and supporting tissue regeneration.

## Conclusions

A chitosan/polyvinyl alcohol (PVA) hydrogel was developed and characterized as a possible gastroretentive delivery system for ranitidine. The formulation showed good physicochemical properties, such as nanoscale particle size, positive zeta potential, high encapsulation efficiency, and sustained drug release. Its swelling capacity, degradation behavior, and stability under simulated gastric conditions suggest it could be suitable for prolonged gastric retention. Initial in vitro and in vivo studies indicated that the hydrogel might improve ranitidine’s antioxidant, anti-inflammatory, and anti-ulcer effects. It also demonstrated acceptable biocompatibility and reduced cytotoxicity compared to the free drug. However, these findings are preliminary and need more validation through detailed pharmacokinetic studies, long-term safety assessments, and thorough in vivo evaluations to confirm the clinical use of the formulation. Despite promising results, this study has limitations that must be recognized. The lack of pharmacokinetic and biodistribution data hinders understanding of the hydrogel’s drug delivery potential. Long-term safety, toxicity, and immune responses were not evaluated, requiring further research. Although the hydrogel was stable in simulated gastric conditions, its long-term shelf life and performance in different environments need additional investigation. Variability in polymer–drug interactions, especially under physiological conditions, could influence drug release and therapeutic results. Addressing these issues is crucial for advancing toward clinical development.

## Data Availability

The datasets of the current study are available from the corresponding author on a reasonable request.
